# Targeting *MALAT1* Augments Sensitivity to PARP Inhibition by Impairing Homologous Recombination in Prostate Cancer

**DOI:** 10.1158/2767-9764.CRC-23-0089

**Published:** 2023-10-09

**Authors:** Anjali Yadav, Tanay Biswas, Ayush Praveen, Promit Ganguly, Ankita Bhattacharyya, Ayushi Verma, Dipak Datta, Bushra Ateeq

**Affiliations:** 1 Molecular Oncology Laboratory, Department of Biological Sciences and Bioengineering, Indian Institute of Technology Kanpur, Kanpur, Uttar Pradesh, India.; 2 Division of Cancer Biology, CSIR-Central Drug Research Institute, Lucknow, Uttar Pradesh, India.; 3 Mehta Family Centre for Engineering in Medicine, Indian Institute of Technology Kanpur, Kanpur, Uttar Pradesh, India.; 4 Centre of Excellence for Cancer - Gangwal School of Medical Sciences and Technology, Indian Institute of Technology Kanpur, Kanpur, Uttar Pradesh, India.

## Abstract

PARP inhibitors (PARPi) have emerged as a promising targeted therapeutic intervention for metastatic castrate-resistant prostate cancer (mCRPC). However, the clinical utility of PARPi is limited to a subset of patients who harbor aberrations in the genes associated with the homologous recombination (HR) pathway. Here, we report that targeting *metastasis-associated lung adenocarcinoma transcript 1* (*MALAT1*), an oncogenic long noncoding RNA (lncRNA), contrives a BRCAness-like phenotype, and augments sensitivity to PARPi. Mechanistically, we show that *MALAT1* silencing reprograms the homologous recombination (HR) transcriptome and makes prostate cancer cells more vulnerable to PARPi. Particularly, coinhibition of *MALAT1* and PARP1 exhibits a decline in clonogenic survival, delays resolution of γH2AX foci, and reduces tumor burden in mice xenograft model. Moreover, we show that miR-421, a tumor suppressor miRNA, negatively regulates the expression of HR genes, while in aggressive prostate cancer cases, miR-421 is sequestered by *MALAT1*, leading to increased expression of HR genes. Conclusively, our findings suggest that *MALAT1* ablation confers sensitivity to PARPi, thus highlighting an alternative therapeutic strategy for patients with castration-resistant prostate cancer (CRPC), irrespective of the alterations in HR genes.

**Significance:**

PARPi are clinically approved for patients with metastatic CRPC carrying mutations in HR genes, but are ineffective for HR-proficient prostate cancer. Herein, we show that oncogenic lncRNA, *MALAT1* is frequently overexpressed in advanced stage prostate cancer and plays a crucial role in maintaining genomic integrity. Importantly, we propose a novel therapeutic strategy that emphasizes *MALAT1* inhibition, leading to HR dysfunction in both HR-deficient and -proficient prostate cancer, consequently augmenting their susceptibility to PARPi.

## Introduction

Prostate cancer is a molecularly heterogeneous and multifocal disease, with each foci exhibiting varying cellular disorganization and molecular alterations. Because of this heterogeneity, comprehending the molecular mechanisms underlying disease progression would help to develop effective screening, preventive, and treatment strategies (1, 2). Advancements in sequencing technologies revealed that prostate cancer progression is accompanied by the acquisition of multiple genomic aberrations, including somatic and/or germline mutations, copy-number alterations, microsatellite variations, and chromosomal alterations such as translocations, duplications, insertions, and deletions. Intriguingly, approximately 20%–30% of patients with advanced-stage prostate cancer harbor genomic aberrations in the genes associated with the DNA damage and repair (DDR) pathway, the most notable being loss-of-function mutations in *BRCA1/2* (3, 4). Mutations in DDR genes alleviate the repair capacity of tumor cells and facilitate complex cancer-related genetic alterations (5). Nevertheless, several recent reports suggest that patients with castration-resistant prostate cancer (CRPC) exhibit higher expression of homologous recombination (HR) genes, including *BRCA1*, *RAD54L*, and *RMI2*, compared with localized cases (6). The mechanism(s) underlying their overexpression and the associated functional consequences are not yet established. Nevertheless, several chemotherapy drugs, including platinum-based medicines (7), radiation (8), and antiandrogens (9) kill cancer cells by inducing DNA damage. Consequently, cells that effectively repair DNA damage can withstand therapeutic drugs. Moreover, rapid DNA damage response activation and enhanced DNA repair capacity have been linked to therapeutic resistance in multiple cancer types (10, 11), suggesting that overexpression of the HR pathway might provide a survival advantage to CRPC cells and facilitate cancer progression.

Alternatively, long noncoding RNAs (lncRNA) have recently been demonstrated to be crucial players in the repair of DNA damage and offer novel targets for combating cancer resistance (12, 13). For instance, antisense RNA in the INK4 locus (*ANRIL*, ref. 14), DNA damage-sensitive RNA1 (*DDSR1*, ref. 15), and lncRNA radiation-induced regulator of PLK1 and RAD51 (*lnc-RI*, ref. 16) have been shown to modulate the DNA repair capacity by enhancing HR. Despite the intricate involvement of lncRNAs in maintaining genome integrity, thus far, only one study in prostate cancer has shown the direct interplay between lncRNAs and the DDR pathway, wherein they demonstrated that *PCAT-1* induces functional deficiency in HR by repressing *BRCA2* (17). Therefore, identifying lncRNAs that modulate genomic stability in prostate cancer and investigating their biological roles can aid in deciphering the mechanisms underlying the development of therapy-resistant prostate cancer.

Here, we unraveled a molecular network involving the lncRNA *MALAT1* and the HR pathway in mCRPC. We showed that *MALAT1* modulates DNA repair pathways and plays an essential role in maintaining genomic integrity in patients with advanced-stage prostate cancer. RNAi-mediated depletion of *MALAT1* induces HR deficiency, which in turn enhances sensitivity to olaparib, a widely used PARP1 inhibitor. Collectively, this study provides strong evidence that *MALAT1* is indispensable for maintaining genome integrity in advanced-stage prostate cancer, emphasizing a novel therapeutic approach wherein targeting *MALAT1* augments sensitivity to PARP inhibition by inducing HR deficiency in prostate cancer.

## Materials and Methods

### *In-Silico* Data Processing and Computational Analysis

#### Microarray Analysis

The gene expression datasets, namely GSE35988 (18), GSE6919 (19), and GSE6752 (20), were downloaded from the GEO database (https://www.ncbi.nlm.nih.gov/geo/, RRID:SCR_005012) each of which comprises expression profiles for benign, localized prostate cancer, and CRPC samples. The differentially expressed genes (DEG) in patients with CRPC were identified using the “limma” package (RRID:SCR_010943) in R (21) with the cut-off criterion of an adjusted *P* value (*P*_adj_) <0.05 and log_2_ fold change |FC| > 0.6. Furthermore, the Venn analysis web tool at https://bioinformatics.psb.ugent.be/webtools/Venn was used to identify the commonly elevated genes in CRPC samples from the three groups. The samples were sorted on the basis of tissue type, and *MALAT1* expression was plotted [log_2_ (normalized count)] using GraphPad Prism version 8.0 (RRID:SCR_002798). No cutoff was applied to the dataset.

#### Integrative Analyses for TCGA-PRAD Data

The HiSeq mRNA data for *MALAT1* and clinical information for the TCGA-PRAD dataset were downloaded from the UCSC Xena browser (https://xenabrowser.net/, RRID:SCR_018938). To analyze the association of *MALAT1* with the primary Gleason score, the samples were divided into three groups corresponding to primary Gleason scores 3, 4, and 5, and the data were plotted for *MALAT1* expression (log_2_ (norm_count+1)). A similar analysis was performed for node status, response to therapy, and biochemical recurrence. For Kaplan–Meier survival analysis (RRID:SCR_021137), survival data of patients with primary tumors were retrieved from the UCSC Xena browser. Days to the first biochemical recurrence and up to the last follow-up were the two parameters considered for analysis. The samples were stratified into two groups based on the median expression value of *MALAT1*, wherein patients with expression values higher than the median were placed in the “*MALAT1* high” group, while patients with expression values lower than the median were placed in the “*MALAT1* low” group. Furthermore, Kaplan–Meier survival analysis was performed using GraphPad Prism 8.0 to calculate the 5-year survival probability.

To examine the association of *MALAT1* with epithelial, mesenchymal, and stemness markers, we arranged the TCGA-PRAD patients’ samples in descending order of *MALAT1* expression and divided the dataset into four “equal quartiles.” The top 25% of the patients (*n* = 125) corresponding to the upper quartile [QU, log_2_ (RPM+1) > 12.29] were assigned as *MALAT1*-high samples, while the lower quartile [QL, log_2_ (RPM+1) < 11.05] were considered *MALAT1*-low samples. The corresponding expression values for epithelial, mesenchymal, and stemness genes in *MALAT1*-high and *MALAT1*-low groups (without further cutoffs) were considered to examine their association with *MALAT1*.

#### Gene Coexpression Analysis

The pairwise Pearson correlation coefficient (*ρ*) between *MALAT1* and DDR/cell-cycle genes in prostate cancer cohorts, namely GSE35988 (18), GSE3325 (22), and GSE77930 (23) was examined using the “corrplot” package (RRID:SCR_023081) in R. *P* < 0.05 was considered as a statistically significant threshold. Also, gene-set variation analysis (GSVA) was performed to investigate the variations in the activation status of the DDR pathway and G_1_–S phase transition in different clinical stages of prostate cancer. The gene sets for the “DDR pathway” and the “G_1_–S phase transition” were downloaded from the MSigDB database (RRID:SCR_016863). Ultimately, an R package named “complex heatmap” (RRID:SCR_017270) was used to demonstrate the enrichment of the genes mentioned above in each group and the expression of *MALAT1* (24).

#### RNA-Sequencing Analysis

The transcriptome profiles for LNCaP-siNT and LNCaP-si*MALAT1* (GSE72534; ref. 25) were downloaded from the sequence read archive (SRA) and analyzed in the galaxy (usegalaxy.org, RRID:SCR_006281). Raw sequencing FASTQ reads were prefetched and Fastq-dumped using the SRA-toolkit (http://ncbi.github.io/sra-tools/, RRID:SCR_004891). FastQC sequence quality checks were performed on the raw reads before mapping them to hg38 human using TopHat v2.1.0 (RRID:SCR_013035). Within the sample, FPKM normalization was performed for both conditions. The downregulated genes in LNCaP-abl-si*MALAT1* cells (log_2_ FPKM difference ≤−0.6) were then subjected to the Database for Annotation, Visualization, and Integrated Discovery (DAVID, RRID:SCR_001881) bioinformatics platform (26) to identify deregulated biological processes (*P* < 0.05).

#### Cancer Cell Line Encyclopedia Analysis

*MALAT1* mRNA expression in various cancer cell lines was retrieved from the Cancer Cell Line Encyclopedia (CCLE) database (RRID:SCR_013836) and was used to analyze its association with cancer type. The samples were sorted on the basis of tissue type, and *MALAT1* expression was plotted using GraphPad Prism version 8.0.

### Experimental Methods

#### Cell Line Culture Conditions and Authentication

The prostate cancer cell lines namely 22RV1 (RRID:CVCL_1045), VCaP (RRID:CVCL_2235), LNCaP (RRID:CVCL_0395), and PC3 (RRID:CVCL_0035) and benign prostate epithelial cells (PNT2, RRID:CVCL_2164) were sourced from the ATCC. DU145 (RRID:CVCL_0105) cells were generously gifted by Dr. Mohammad Asim, Department of Clinical and Experimental Medicine, Faculty of Health and Medical Sciences, University of Surrey (Guildford, United Kingdom). The cells were cultured according to ATCC-recommended guidelines in 37°C incubators with 5% CO_2_. Cell lines were typically cultured no longer than continuous 45 days upon thawing the frozen vials, and were grown up to 25 passages. Cell lines were periodically tested for *Mycoplasma* contamination (last tested: January 2021) using the PlasmoTest Mycoplasma Detection Kit (InvivoGen). The cell lines used in this study were authenticated by short tandem repeat profiling at the Life Code Technologies Private Limited, and DNA Forensics Laboratory (last authentication: December 2017).

#### Lentiviral Packaging

ViraPower Lentiviral Packaging Mix (Invitrogen) was used to generate viral particles for shSCRM and sh*MALAT1* (Supplementary Table S1) as previously described (27). Briefly, the shRNA constructs and packaging mix plasmids were transfected into HEK293FT (RRID:CVCL_6911) cells and incubated for 60–72 hours. The viral particles were then harvested and stored at −80°C for long-term storage. To create stable knockdown cell lines, the prostate cancer cells were infected with the collected lentiviral particles along with polybrene (hexadimethrine bromide; 8 µg/mL; Sigma-Aldrich). The culture medium was changed 24 hours after infection, and puromycin (Sigma-Aldrich) selection was started 72 hours later.

#### CRISPR-Cas9–Based *MALAT1* Knockout

Lentiviral vectors containing a pair of guide RNAs (gRNA) targeting human *MALAT1* (pDECKO_*MALAT1*_C, RRID:Addgene_72622, ref. 28), control gRNAs in pDECKO_GFP (RRID:Addgene_72619), and Cas9 (lentiCas9-Blast, RRID:Addgene_52962, ref. 29) were procured from Addgene, and lentiviral particles were produced. 22RV1 cells were then infected with the lenti-Cas9-Blast lentivirus and selected with blasticidin (5 µg/mL). Cas9-overexpressing cells were infected with the pDECKO_GFP and pDECKO_*MALAT1*_C lentiviruses and selected with puromycin (2 µg/mL). Single cells from a pooled population of pDECKO_*MALAT1*_C were injected into each well of a 96-well plate and cultured for another 2–3 weeks under puromycin selection to obtain single clones with *MALAT1* knockout. For knockout validation, the isolated single clones were subjected to genomic PCR and qPCR (Supplementary Table S1).

#### HR Assay

*MALAT1* knockout and control prostate cancer cells were nucleofected with 0.7 µg of pDR-GFP plasmid (RRID:Addgene_26475; ref. 30). Simultaneously *RAD51*-silenced and control prostate cancer cells were also nucleofected with 0.7 µg of pDR-GFP plasmid. Two days later, the cells were transfected with 2 µg of pCBASce (RRID:Addgene_26477; ref. 31). The cells were harvested 48 hours after transfection, and GFP expression was measured using flow cytometry. The percentage of GFP-positive cells was quantified to estimate the number of cells undergoing HR.

#### Chromatin Isolation by RNA Purification

The chromatin isolation by RNA purification (ChIRP) samples were prepared as described by Chu and colleagues (32). Briefly, 22RV1 cells were cross-linked using 1% formaldehyde for 10 minutes at room temperature. Next, formaldehyde was quenched using 125 mmol/L glycine for 5 minutes at room temperature. The cross-linked cells were washed twice with PBS and later lysed in lysis buffer consisting of Tris-Cl (50 mmol/L, pH 7.0), EDTA (10 mmol/L), and 1% SDS. Furthermore, the samples were sonicated to obtain DNA fragments of ∼500bp using the Bioruptor (Diagenode). Subsequently, a cocktail of biotin-labeled probes (25 pmol each; Supplementary Table S1) specific to *MALAT1* was added to the fragmented chromatin and incubated for 4 hours at 37°C in a hybridization chamber. The hybridized content was then retrieved using streptavidin magnetic beads (Dynabeads Streptavidin, Invitrogen). A fraction of the sample was used for RNA extraction to validate *MALAT1* pull-down by qRT-PCR, while the rest of the sample was used for protein sample preparation.

#### Mice Xenograft Study

All experimental procedures using mice were approved by the Committee for the Purpose of Control and Supervision of Experiments on Animals (CPCSEA) and abided by all regulatory standards of the Institutional Animal Ethics Committee of the Indian Institute of Technology Kanpur. Five- to 6-week-old male NOD.CB17-Prkdcscid/J (NOD/SCID; Jackson Laboratory, RRID:BCBC_4142) mice were anesthetized using a cocktail of ketamine and xylazine (50 mg/kg and 5 mg/kg, respectively) injected intraperitoneally. 22RV1-*MALAT1*-KO and 22RV1-SCR control cells were trypsinized and resuspended (2.5 × 10^6^) in 100 µL of saline with 20% Matrigel and implanted subcutaneously at the dorsal flank on both sides of mice (*n* = 12 for each condition). On alternate days, tumor burden was measured with a digital Vernier caliper and tumor volume was calculated using the formula (π/6) (*L* × *W*^2^), (*L* = length; *W* = width). When the average tumor volume reached 100 mm^3^, the mice in each group were randomized into two groups (*n* = 6) and treated five times a week with either vehicle control or olaparib (50 mg/kg) diluted in 5% dimethyl sulfoxide (DMSO), 20% polyethylene glycol 400 (PEG-400; Sigma Aldrich) by oral gavage. To investigate spontaneous metastases to lungs and bone marrow of the xenografted mice, genomic DNA was isolated from these organs and assayed for the expression of human Alu-elements using TaqMan probe FAM-YB8-ALU-167 with sequence 5′-6-FAM-AGCTACTCGGGAGGCTGAGGCAGGA-TAMRA-3′, which specifically hybridizes to the human-specific YB8-Alu sequences (33). The standard curve for the TaqMan assay was generated by using serially diluted human genomic DNA spiked with mouse genomic DNA, and based on the *C*_t_ values, the number of metastasized cells was calculated.

#### IHC of Tumor Xenografts

Following the removal of tumor tissues from xenografted mice, they were fixed in 10% buffered formalin, paraffin-embedded, and sectioned at 3-µm thickness using a microtome (Leica). After tissue sections were deparaffinized and rehydrated, sodium citrate buffer was used for heat-induced antigen retrieval (pH 6.0). Furthermore, endogenous peroxidase activity was quenched with 3% hydrogen peroxide (H_2_O_2_) and blocked using 5% normal goat serum. The tissue sections were incubated with the Ki-67 (RRID:AB_2797703) antibody at 4°C overnight. Next morning, the sections were first washed with Tris-buffered saline (TBS) and then incubated with a biotinylated secondary antibody at room temperature for 1 hour, followed by incubation with an ABC (avidin–biotin complex, Vector Laboratories) solution for 30 minutes. Sections were then processed for detection of horseradish peroxidase (HRP) activity using 3, 3-diaminobenzidine (DAB) peroxidase (HRP) substrate kit (Vector Laboratories). Quantification for Ki-67 was performed using ImageJ software using 10 random histologic fields.

### Statistical Analysis

For statistical analysis, unpaired two-tailed Student *t* test, one-way ANOVA, or two-way ANOVA were used unless otherwise specified in the respective figure legend. *P* ≤ 0.05 was considered significant. The error bars indicate SEM calculated from three independent experiments performed in triplicate.

### Data Availability Statement

We have not performed global gene expression profiling or RNA-sequencing (RNA-seq) for this project nevertheless various publicly available datasets were used in this study, namely UCSC Xena (https://xenabrowser.net/) to retrieve the TCGA-PRAD dataset and GEO (www.ncbi.nlm.nih.gov/geo) to retrieve gene expression profiling in patients with prostate cancer: GSE35988, GSE6919, GSE6752, GSE3335, and GSE77930 while RNA-seq data for LNCaP-abl-siNT and -si*MALAT1* was retrieved from GSE72534.

## RESULTS

### Higher *MALAT1* Expression Associates with Advanced stage Prostate Cancer and Poor Prognosis

**FIGURE 1:**
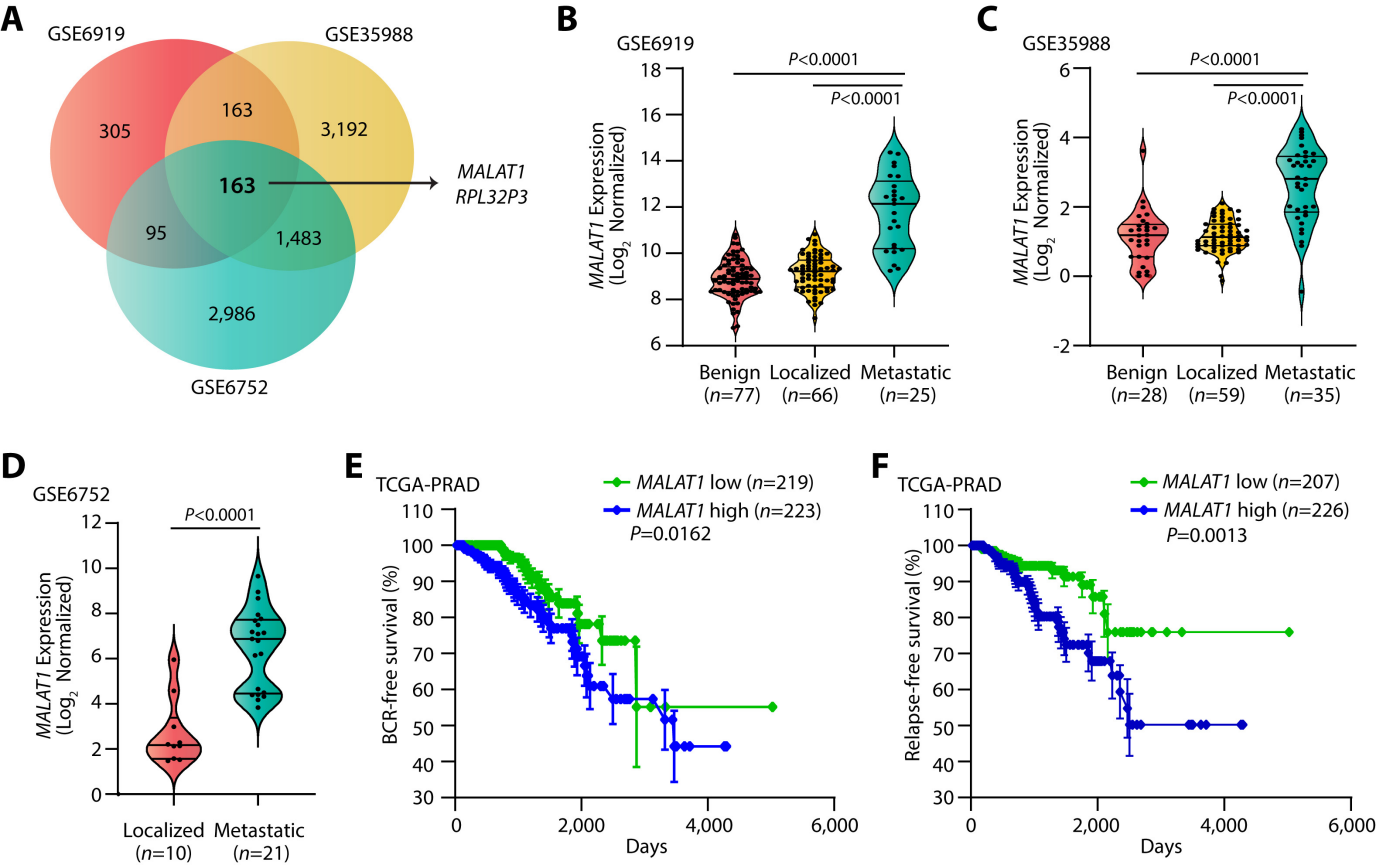
High *MALAT1* expression is associated with poor prostate cancer prognosis. **A,** Venn diagram displaying genes upregulated in patients with metastatic prostate cancer compared with localized cases in three publicly available gene expression omnibus (GEO) datasets namely, GSE35988, GSE6919, and GSE6752. **B,** Dot plot with superimposed violin plot showing *MALAT1* expression in patients with benign, primary, and metastatic prostate cancer in the GSE6919 dataset. *MALAT1* transcript is reported as log_2_ median-centered ratio. **C,** Same as B, except GSE35988 dataset. **D,** Same as B, except GSE6752 dataset. **E,** Kaplan–Meier curves for BCR-free survival in the TCGA-PRAD dataset categorized as “*MALAT1*-high” (*n* = 223) and “*MALAT1*-low” (*n* = 219) groups based on the median expression of *MALAT1*. Blue line represents patients with higher expression of *MALAT1* whereas the green line represents cases with patients with lower expression of *MALAT1*. **F,** Same as E, except relapse-free survival for the TCGA-PRAD dataset. Data represent mean ± SEM. For B and C, one-way ANOVA with Tukey multiple comparison test was applied, while for D, two-tailed unpaired Student *t* test was applied. The *P* values for E and F were computed by the log-rank test.

To identify the essential genes associated with advanced stage prostate cancer, we performed differential gene expression analysis using three publicly available microarray datasets comprising expression profiles of patients with prostate cancer, namely GSE35988 (18), GSE6919 (19), and GSE6752 (20). A total of 163 transcripts comprising 161 genes, a pseudogene (*RPL32P3*), and an oncogenic lncRNA (*MALAT1*) were found to be significantly elevated in patients with metastatic prostate cancer compared with localized cases (Fig. 1A; Supplementary Table S2). We observed that *MALAT1* was highly upregulated in metastatic cases (∼1.5–4 times) compared to patients with localized prostate cancer (Fig. 1B–D). Analysis of transcriptome data from The Cancer Genome Atlas-Prostate Adenocarcinoma (TCGA-PRAD) cohort (*n* = 499; ref. 34) revealed that *MALAT1* expression positively correlates with several clinicopathologic parameters such as Gleason score and node status (Supplementary Fig. S1A). Moreover, elevated levels of *MALAT1* were observed in patients with progressive disease compared with those with complete response after primary therapy (Supplementary Fig. S1A), indicating its significance in predicting response to chemotherapy. Besides, higher *MALAT1* levels were also noted in the patients who suffered from biochemical recurrence (BCR; Supplementary Fig. S1B). To further validate the same, we performed a Kaplan–Meier survival analysis by stratifying the TCGA-PRAD patients based on their *MALAT1* median expression into high (≥median, *n* = 248) and low (≤median, *n* = 250) groups. Intriguingly, patients with elevated *MALAT1* levels showed a higher probability of BCR compared with the *MALAT1*-low group (*P* = 0.0162, Fig. 1E). In addition, *MALAT1*-high patients in the TCGA-PRAD cohort showed higher chances of relapse compared with the *MALAT1*-low group (*P* = 0.0013, Fig. 1F), suggesting that *MALAT1* levels could also predict the likelihood of disease recurrence.

RNA-seq data analyses from the CCLE revealed high expression of *MALAT1* in multiple human cancer cell lines, with the highest expression noted in prostate cancer (Supplementary Fig. S1C). Similarly, our quantitative PCR (qPCR) data with multiple prostate cancer cell lines (PC3, DU145, LNCaP, 22RV1, and VCaP) also exhibited higher expression of *MALAT1* compared with PNT2, an immortalized nontumorigenic normal prostate epithelial cell line (Supplementary Fig. S1D). Taken together, these results indicate that *MALAT1* positively correlates with the clinicopathologic features of aggressive prostate cancer and may serve as a promising prognostic marker.

### *MALAT1* Promotes Epithelial-to-Mesenchymal Transition, Stemness, and Chemoresistance in Prostate Cancer

**FIGURE 2:**
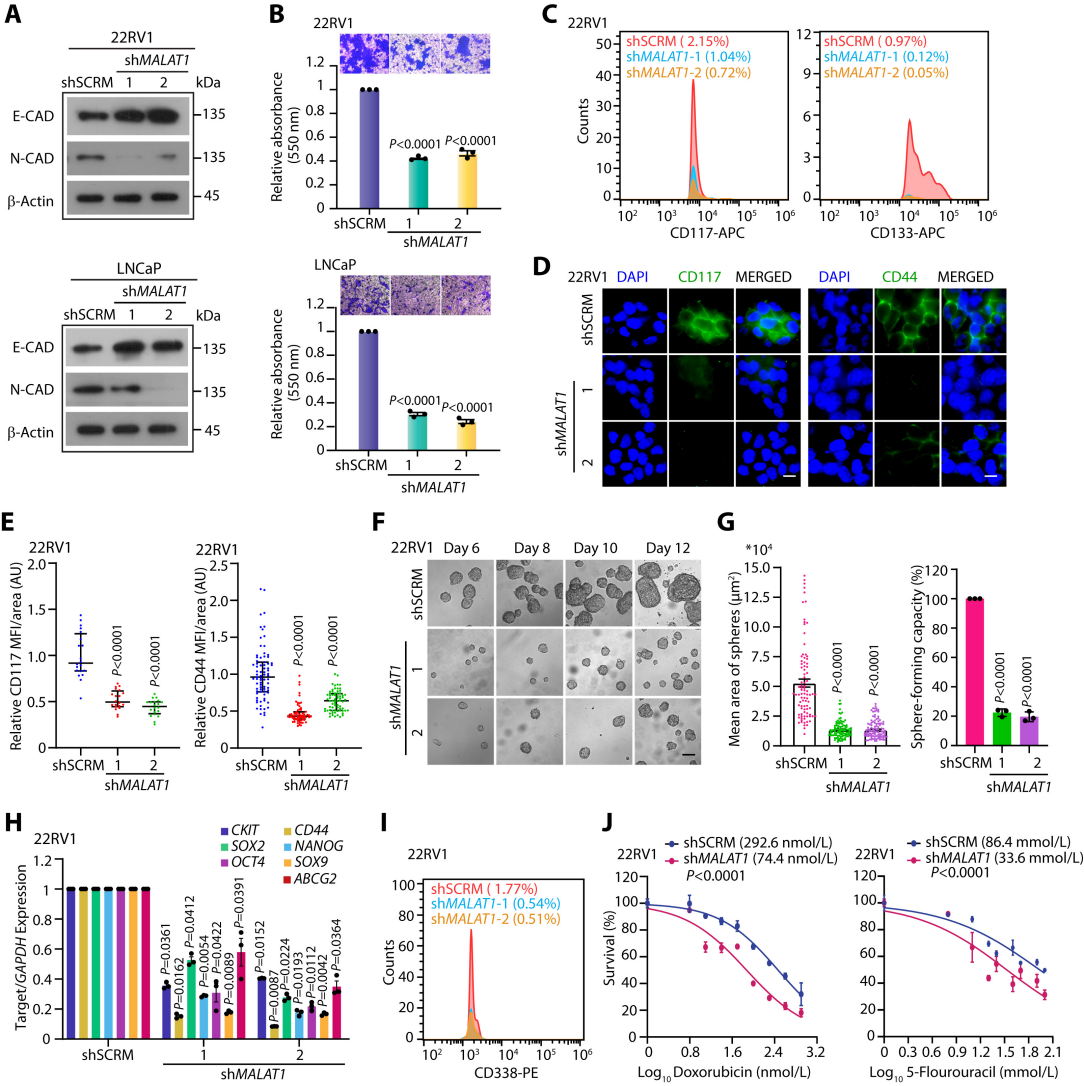
*MALAT1* promotes EMT, stemness, and chemoresistance in prostate cancer. **A,** Immunoblots showing the expression of EMT markers in sh*MALAT1* and shSCRM prostate cancer cells. β-Actin was used as an internal control. **B,** Boyden chamber Matrigel migration assay using the same cells as in A. Representative fields with the migrated cells are shown in the inset. The bar plot depicts the alteration in migratory potential of the prostate cancer cells upon *MALAT1* knockdown. **C,** Flow cytometry analysis showing expression of CD117 (c-KIT) and CD133 in 22RV1-sh*MALAT1* and shSCRM control cells. **D,** Immunofluorescence images displaying the expression of CD117 and CD44 and in the same cells as in C. Scale bar, 20 µm. **E,** Dot plot represents quantification of CD117 and CD44 mean fluorescence intensity (MFI) per unit area shown as arbitrary units (AU). **F,** Representative phase-contrast images for the prostatospheres formed using 22RV1-sh*MALAT1* and shSCRM control cells on the indicated days. Scale bar, 100 µm. **G,** Bar plot superimposed with dots represents the mean area of the prostatospheres and percentage sphere formation efficiency. **H,** qPCR depicting the expression of stem cell markers in the prostatospheres derived from the same cells as in E. Expression level for each gene was normalized to *GAPDH*. **I,** Flow cytometry analysis showing expression of ATP-binding cassette superfamily G member 2 (CD338/ABCG2) using the same cells as in C. **J,** Cell cytotoxicity assay using chemotherapeutic drugs namely, doxorubicin and 5-fluorouracil using the same cells as in C. IC_50_ values were calculated by generating a dose–response curve using GraphPad Prism software. Experiments were performed with *n* = 3 biologically independent samples; the data represents mean ± SEM. The statistical difference among the groups was computed using one-way ANOVA with Dunnett multiple-comparison test for B, E, G, and H.

*MALAT1* has emerged as a metastasis-associated lncRNA in multiple malignancies and serves as a predictor in assessing response to cancer therapies (35–37). As indicated in Fig. 1, tumors with elevated levels of *MALAT1* also show a higher propensity for lymph node metastasis, disease recurrence, and therapeutic failure in patients with prostate cancer. Therefore, we examined the association of *MALAT1* with molecular factors allied with epithelial-to-mesenchymal transition (EMT) in the TCGA-PRAD cohort. Intriguingly, *MALAT1*-low patients showed increased levels of archetypal epithelial markers, such as *CHD1*, *EPCAM*, *TJP1*, *CLDN7*, and *OCLN* compared with *MALAT1*-high patients (Supplementary Fig. S2A). Concomitantly, patients with higher expression of *MALAT1* showed elevated levels of mesenchymal markers, such as *CTGF*, *KRT5*, *FOXC1*, *SNAI1*, and *FOXC2* (Supplementary Fig. S2B). To further investigate the functional significance of *MALAT1* in EMT, we generated stable *MALAT1*-silenced (sh*MALAT1*) and scrambled control (shSCRM) 22RV1 and LNCaP cells using lentivirus-based short hairpin RNAs (shRNA; Supplementary Fig. S2C and S2D). Characterization of these stable cells revealed a robust increase in the epithelial marker, E-cadherin, with a concomitant decrease in the mesenchymal marker (N-cadherin) in sh*MALAT1* cells compared with respective shSCRM control, emphasizing its importance in EMT (Fig. 2A). In accord with this, *MALAT1* depletion remarkably reduced 3D cell migration in 22RV1 (∼60%) and LNCaP (∼70%) cells compared with shSCRM controls (Fig. 2B), suggesting that *MALAT1* is required for prostate cancer cell migration. Metastatic prostate cancer cells frequently acquire cancer stem cell (CSC) properties, which result in self-renewal and chemoresistance (38, 39), therefore we examined the TCGA-PRAD cohort for any association of *MALAT1* with self-renewal factors. Interestingly, *MALAT1*-high patients showed increased levels of key stemness factors, such as *OCT4*, *NANOG*, *KLF4*, ATP-binding cassette subfamily G member 2 (*ABCG2*), sex-determining region Y-Box 9 (*SOX9*) compared with *MALAT1*-low patients (Supplementary Fig. S2E), indicating a positive association of *MALAT1* with stemness. To confirm this, we evaluated the expression of a few cell surface markers associated with CSCs, namely CD117 (c-KIT), a tyrosine kinase receptor; CD133 (prominin-1); and CD44 (HCAM), a cell-surface glycoprotein, in 22RV1-sh*MALAT1* and -shSCRM cells. Interestingly, sh*MALAT1* cells displayed a marked reduction in the expression of CD117 (∼50–70%), CD133 (∼90%), and CD44 (∼80%) compared with shSCRM control (Fig. 2C–E). Because self-renewal is an essential feature of stemness, we next examined the ability of 22RV1-sh*MALAT1* and -shSCRM cells to form prostatospheres. As anticipated, *MALAT1* depletion abrogated the prostatosphere-forming ability of 22RV1 cells as well as their expansion in subsequent serial propagations (Fig. 2F and G). Moreover, the prostatospheres formed with *MALAT1-*deficient cells were significantly smaller compared with the SCRM control (Fig. 2G). Molecular characterization of 22RV1-sh*MALAT1* prostatospheres showed a significant reduction in the expression of pluripotency genes, namely *C-KIT*, *OCT-4*, *NANOG*, *CD44*, *SOX2/9*, and *ABCG2* (Fig. 2H). Likewise, a robust decrease in the expression of CD338 (ABCG2), an ATP-binding cassette transporter, was also noted in 22RV1 sh*MALAT1* cells compared with 22RV1-shSCRM (Fig. 2I), suggesting that *MALAT1* modulates stemness in prostate cancer cells. Aside from stem cell maintenance, several pluripotency factors, including CD117 and ABCG2, have been shown to confer chemotherapeutic resistance. In line with this, Liu and colleagues reported that a subpopulation of the 22RV1 cells that abundantly expressed CD117 and ABCG2; exhibited multidrug resistance (40). Hence, we next investigated the susceptibility of *MALAT1*-silenced cells to chemotherapeutic drugs, namely doxorubicin and 5-fluorouracil (5-FU). Interestingly, 22RV1-sh*MALAT1* cells exhibited enhanced sensitivity to chemotherapeutic drugs compared with 22RV1-shSCRM (Fig. 2J). These findings, thus, provide compelling evidence that the downregulation of *MALAT1* effectively suppresses EMT as well as stemness and confers sensitivity to chemotherapeutic agents.

### *MALAT1* Modulates DNA Repair Pathways and Maintains Genome Integrity in Metastatic Prostate Cancer

**FIGURE 3:**
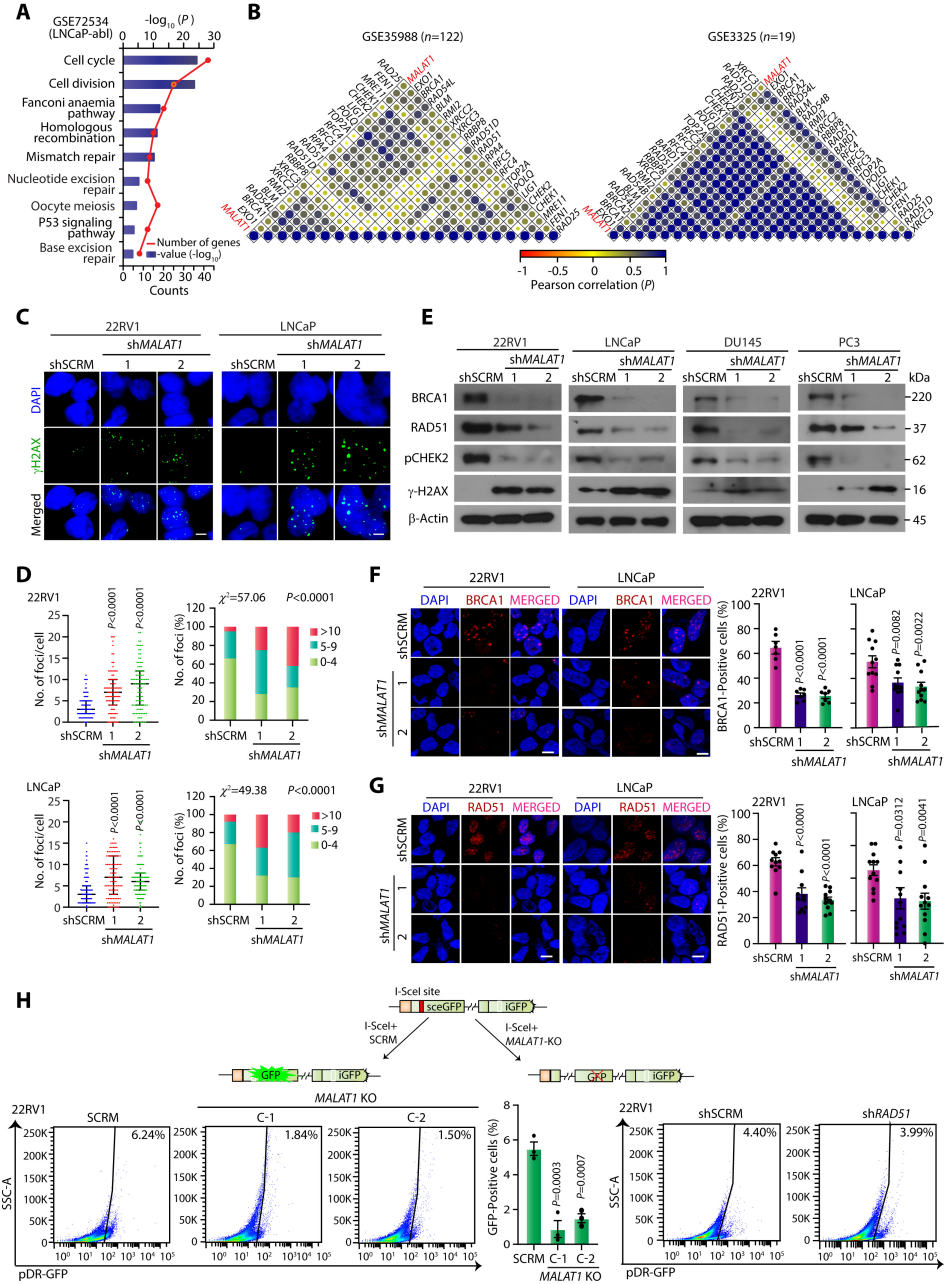
*MALAT1* depletion impairs HR-mediated DSB repair in prostate cancer cells. **A,** DAVID analysis depicting biological pathways downregulated in LNCaP-abl-si*MALAT1* cells relative to LNCaP-abl–siCTL. Bars represent -log_10_ (*P*) and the frequency polygon (line in orange) represents the number of genes. **B,** Correlogram depicting Pearson correlation coefficient (*ρ*) between DNA repair–associated genes and *MALAT1* in prostate cancer patient samples from GSE35988 and GSE3325 datasets (FDR adjusted, *P* < 0.05). Correlation coefficients are expressed by the color from red to blue and the dot size is proportional to the strength of the correlation. Representative genes are marked on the sides of the correlogram. **C,** Representative confocal images for γH2AX foci (green) in control and *MALAT1*-ablated 22RV1 and LNCaP cells. The nucleus was visualized by DAPI (blue). Scale bar, 10 µm. **D,** Quantification of the number of γH2AX-positive foci in the indicated cells. Bar plot showing the percentage of cells with the indicated number of foci/nuclei in the same cells. The *P* value for the χ^2^ test is indicated. **E,** Immunoblots showing the expression of HR markers in *MALAT1*-silenced and shSCRM prostate cancer cells. β-Actin was used as a loading control. **F,** Same as C, except immunostaining for BRCA1. **G,** Same as C, except immuno-staining for RAD51. **H,** Schematic of the pDR-GFP reporter used to monitor HR activity in 22RV1 *MALAT1*-KO and shRAD51 cells. Bar plot exhibiting the percentage of GFP^+^ cells in 22RV1-*MALAT1*-KO and shRAD51 cells transfected with pDR-GFP reporter construct. Experiments were performed with *n* = 3 biologically independent samples; the data represent mean ± SEM. For D and F–H, one-way ANOVA with Dunnett multiple comparisons *posthoc* test was applied while the χ^2^ test was used for D.

To further delve into the molecular mechanism(s) underlying *MALAT1-*mediated carcinogenesis in the prostate gland, we analyzed the transcriptome profiles of *MALAT1-*silenced and control LNCaP-abl cells (LNCaP-derived castration-resistant cells) retrieved from a publicly available dataset, GSE72534 (25). Our analysis revealed 4,139 differentially expressed transcripts with a log_2_ FPKM difference (≤−0.6 or ≥0.6) that include 1,986 up- and 2,153 downregulated genes in *MALAT1-*silenced LNCaP-abl cells compared with the control cells. Functional annotation of the differentially downregulated genes using DAVID demonstrates several pathways associated with DDR, cell division, and cell cycle as the most significantly downregulated biological processes upon depletion of *MALAT1* in LNCaP-abl cells (Fig. 3A; Supplementary Table S3). Consistent with these findings, GSVA using three prostate cancer cohorts (GSE35988, GSE3325, and GSE77930) exhibited significant enrichment (FDR < 0.05) of the gene signatures associated with DDR and G_1_-S phase transition in metastatic prostate cancer (Supplementary Fig. S3A). Notably, a strong positive correlation (*ρ* ≥ 0.35) between *MALAT1* expression and the DDR gene signature (retrieved from mSigDB) was also observed in these prostate cancer datasets (Fig. 3B; Supplementary Fig. S3B). To examine the clinical significance of *MALAT1*-associated DDR genes, we performed Kaplan–Meier survival analysis for recurrence-free survival using the RNA-seq data of patients with prostate cancer from the TCGA-PRAD cohort categorized into two groups based on the median expression of *MALAT1* and selected DDR genes. Intriguingly, patients with *MALAT1*-low and DDR-low signatures showed higher recurrence-free survival probability compared with *MALAT1*-high and DDR-high patients (Supplementary Fig. S3C).

We next examined the frequency of double-strand breaks (DSB) in *MALAT1*-silenced cells by evaluating the phosphorylation of H2AX on serine 139 residue (γH2AX). A marked increase in the abundance of γH2AX foci was observed in *MALAT1-*silenced 22RV1 and LNCaP cells (Fig. 3C and D), indicating that loss of *MALAT1* results in the accumulation of damaged lesions. Notably, RNA-seq data of *MALAT1-*depleted LNCaP-abl cells show a marked decrease in the expression of genes that encode proteins involved in the HR pathway (Supplementary Fig. S3D). Likewise, 22RV1-sh*MALAT1* and LNCaP-sh*MALAT1* cells showed a significant reduction in the expression of major DNA damage effector proteins, including RAD51 and breast cancer gene 1/2 (BRCA1/2) both at the transcript (Supplementary Fig. S3E) and protein levels (Fig. 3E–G). Notably, a robust decrease in phosphorylation of CHEK2, a central kinase involved in HR signaling, was also observed in *MALAT1-*silenced 22RV1 and LNCaP cells (Fig. 3E). Because both 22RV1 and LNCaP cells harbor inactivating mutations in *BRCA1/2* genes, we next examined whether *MALAT1* can modulate the HR pathway in HR-proficient prostate cancer cells. Hence, we silenced *MALAT1* using specific shRNAs in DU145 and PC3 cells (Supplementary Fig. S3F and S3G) and examined the expression of HR proteins. Interestingly, *MALAT1* silencing led to reduced expression of RAD51, BRCA1, and BRCA2 (Fig. 3E). Concomitantly, a marked increase in the phosphorylation of H2AX foci was also noticed in *MALAT1*-silenced DU145 and PC3 cells (Fig. 3E), indicating the crucial role of *MALAT1* in modulating the expression of several HR genes as well as genome integrity in both HR-proficient and -deficient prostate cancer cells.

To ascertain that *MALAT1* modulates DNA repair by regulating HR activity, we sought to perform the direct repeat-GFP (DR-GFP) reporter assay, a prototypic assay to measure HR (30). In this vector system, a 24-bp I-*SceI* recognition site is integrated into the *GFP* gene that disrupts the open-reading frame (ORF), and a truncated GFP gene fragment with the correct ORF is inserted downstream in the construct. HR-mediated repair of the cleaved I-*SceI* site employing the downstream fragment would lead to a functional GFP with fluorescence, which could be measured by flow cytometry. Because the shRNAs used in this study are GFP positive and cannot be used for this experiment, CRISPR/Cas9–mediated gene knockout of *MALAT1* was performed in 22RV1 cells (Supplementary Fig. S3H). Two independent *MALAT1*-KO clones with significantly reduced *MALAT1* expression (Supplementary Fig. S3H) along with positive control 22RV1-sh*RAD51* cells were selected for the DR-GFP reporter assay. *MALAT1* ablation resulted in a significant decrease in the GFP signal compared with the control 22RV1 cells (Fig. 3H), implying that *MALAT1* ablation suppresses the HR pathway in shRNA.

### *MALAT1* Silencing Restrains Cell-Cycle Progression and Instigates Apoptosis In Prostate Cancer

**FIGURE 4:**
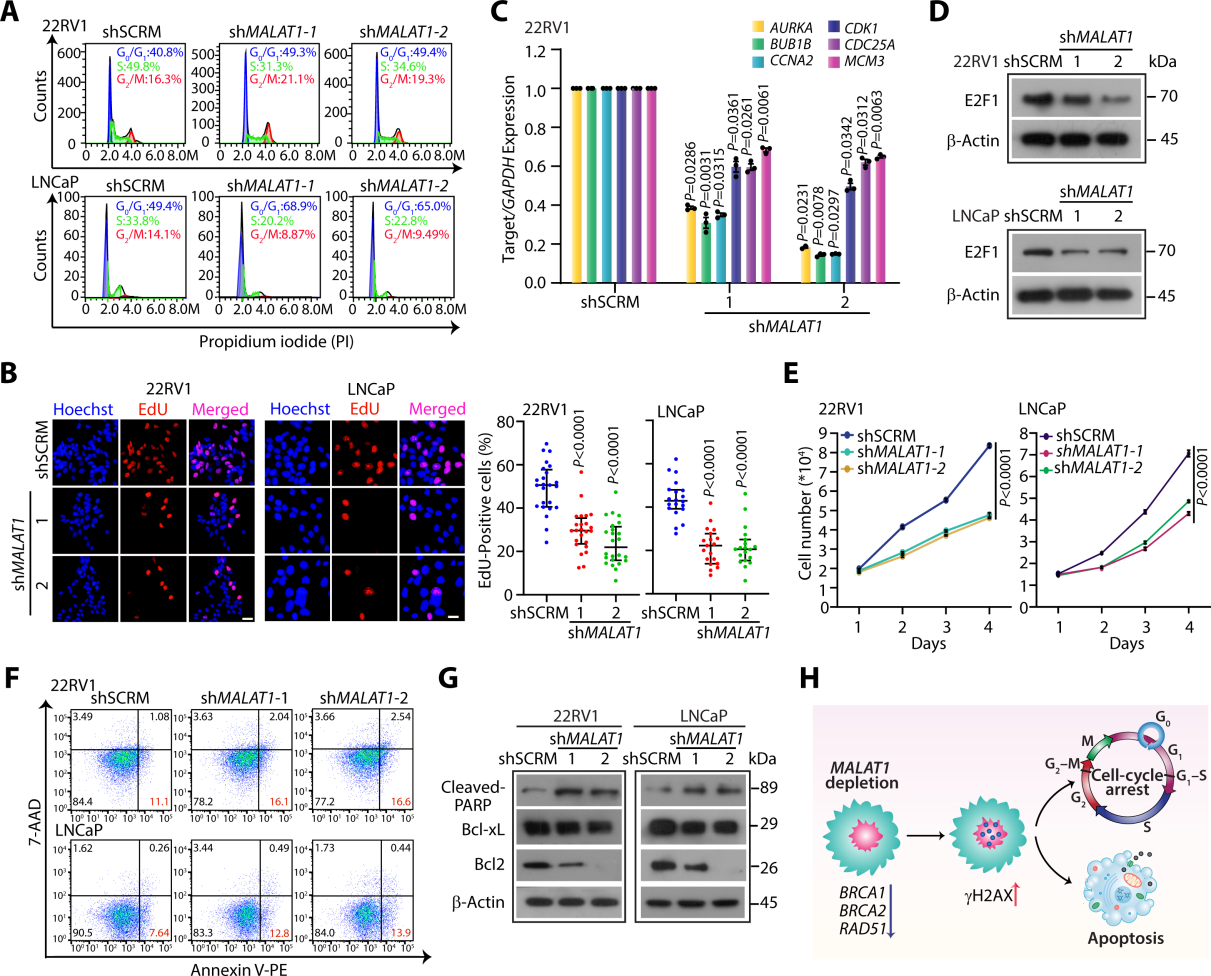
*MALAT1* knockdown restrains cell-cycle progression and instigates apoptosis in prostate cancer cells. **A,** Flow cytometry analysis for accessing the cell-cycle distribution by propidium iodide (PI) DNA staining assay in *MALAT1*-silenced prostate cancer cells. The percentage of cells in each phase was calculated using FlowJo software. **B,** Representative images depicting EdU incorporation in the same cells as in A. Nuclei were stained with Hoechst 33342. Scale bar, 20 µm. Right, bar graph showing quantification of EdU uptake in the indicated cells. **C,** qRT-PCR analysis showing expression of genes associated with G_1_ and S-phase of the cell cycle in *MALAT1*-silenced 22RV1 cells. The expression level for each gene was normalized to *GAPDH*. **D,** Immunoblot showing the change in expression of E2F1 in the same cells as in A. β-Actin was used as an internal control. **E,** Line graph showing cell proliferation assay using the same cells as in A, at the indicated time points. **F,** Flow cytometry–based apoptosis assay using Annexin V-PE and 7-AAD staining in the same cells as in A. The percentage of apoptotic cells was calculated using FlowJo software. **G,** Immunoblots showing a change in the expression of apoptosis markers in the same cells as in A. β-Actin was used as an internal control. **H,** Schematic depicting that *MALAT1* is a novel regulator of HR and plays an important role in the maintenance of genome stability in prostate cancer. *MALAT1* depletion induces HR deficiency by decreasing the expression of several DDR genes and results in DSB accumulation which in turn induces cell-cycle arrest and instigates apoptosis. Experiments were performed with *n* = 3 biologically independent samples; the data represents mean ± SEM. For B and C, one-way ANOVA with Dunnett multiple comparisons *posthoc* test was applied, while for E, two-way ANOVA with Tukey multiple comparisons test was applied.

On accumulation of DSBs, the cellular homeostatic mechanisms either obstruct the cell cycle to give cells more time to repair the lesions or initiate apoptosis if the damage is irreparable (41). Several HR proteins, like RAD51 and BRCA1, exhibit higher expression in the S or G_2_ phase, suggesting that a decrease in the expression of these HR proteins might influence cell-cycle progression (42, 43). Besides, phosphorylation of CHEK2, a critical player regulating the cell-cycle checkpoint, also decreased on *MALAT1* knockdown (Fig. 3E). Moreover, Vidisha and colleagues previously demonstrated that *MALAT1* depletion perturbs the cell-cycle machinery by suppressing the expression of genes involved in G_1_–S and mitotic progression (44). We, therefore, examined the cell-cycle distribution profile by performing propidium iodide (PI) staining. Intriguingly, a robust increase in the G_1_ cells with a concomitant reduction in the S-phase population was noted in *MALAT1-*silenced 22RV1 and LNCaP cells (Fig. 4A). Likewise, the EdU incorporation assay revealed a robust decrease in the number of S-phase cells upon *MALAT1* depletion in 22RV1 and LNCaP cells (Fig. 4B). Intriguingly, the transcriptome profiles of *MALAT1-*silenced LNCaP-abl cells exhibited a marked decrease in the expression of key cell cycle–associated genes (Supplementary Fig. S4A). Corroborating with this, the expression of genes encoding for the proteins associated with G_1_–S and G_2_–M phase transition, such as *CCNA2*, *CCNB*, CDKs, centromere proteins, and mini-chromosome maintenance (MCM2–8) was significantly decreased in *MALAT1-*silenced prostate cancer cells in comparison with shSCRM cells (Fig. 4C; Supplementary Fig. S4B). Similarly, a strong positive correlation (*ρ* ≥ 0.4) between *MALAT1* and cell cycle–associated genes was noted in prostate cancer patient specimens (GSE35988 and GSE3325; Supplementary Fig. S4C), suggesting that *MALAT1* plays a pivotal role in cell-cycle progression. We next examined the expression of E2F1, the major determinant of the G_1_–S phase transition. Notably, a remarkable decrease in E2F1 levels was observed upon *MALAT1* depletion in prostate cancer cells (Fig. 4D). In consonance with this, *MALAT1* ablation markedly reduced cell proliferation in both 22RV1 and LNCaP cells (Fig. 4E).

Because *MALAT1* depletion dysregulates the cellular repair machinery, we speculated that the accumulation of DNA lesions might instigate apoptosis. Thus, we performed annexin-V and 7-AAD staining and noticed a robust increase in early apoptotic cells in *MALAT1-*depleted prostate cancer cells (Fig. 4F). Likewise, levels of cleaved PARP, an early hallmark of apoptosis, also increased upon silencing *MALAT1* in both the cell lines (Fig. 4G). A robust decrease in the antiapoptotic Bcl-2 family proteins (BCL2 and Bcl-xL) was also observed (Fig. 4G), establishing that the loss of *MALAT1* triggers apoptosis. Collectively, these findings establish that *MALAT1* acts as a master regulator that modulates HR, cell-cycle progression, and apoptosis in prostate cancer (Fig. 4H).

### *MALAT1* Modulates the Expression of HR Genes by Sponging miRNA-421

**FIGURE 5:**
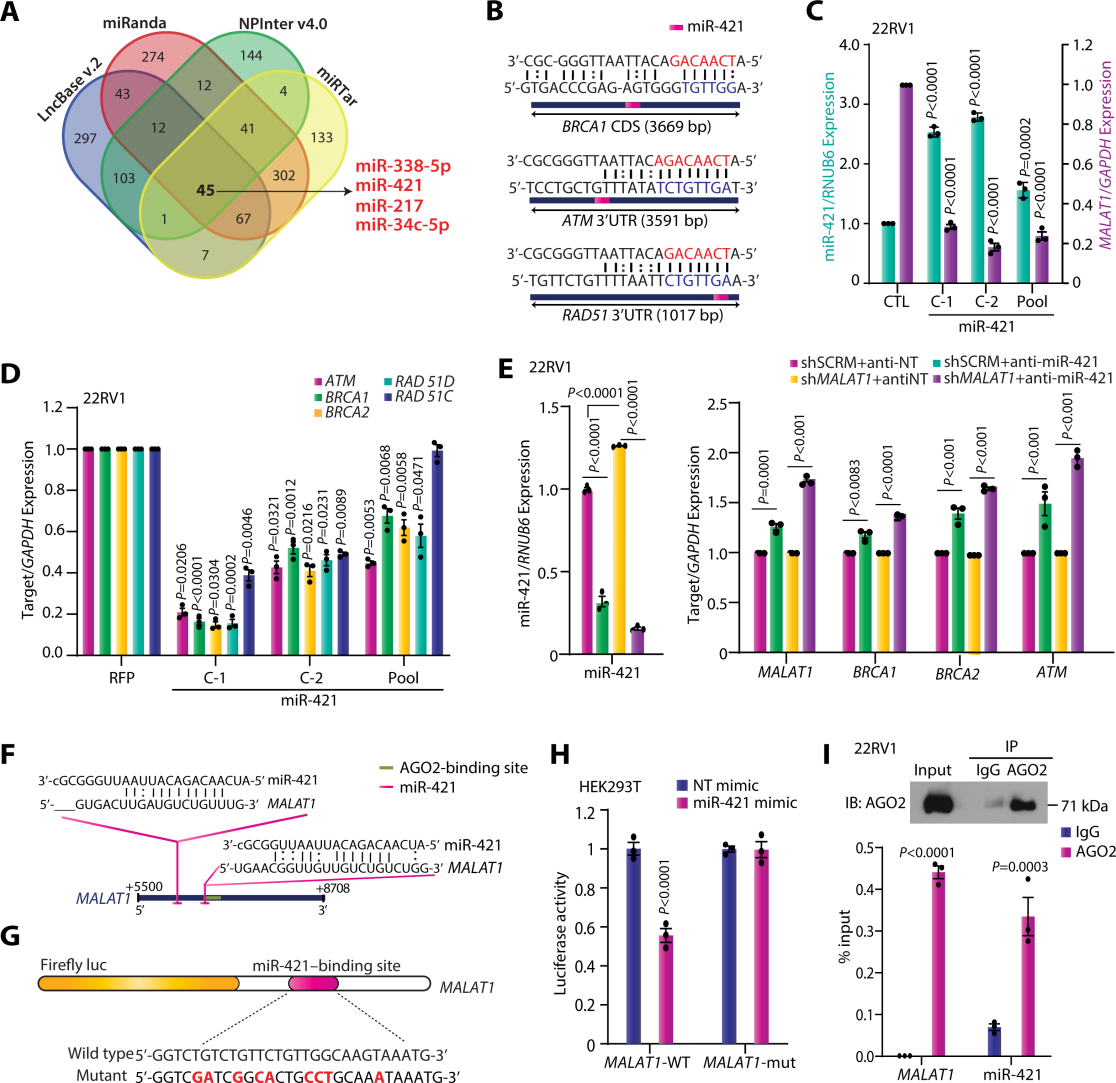
*MALAT1* modulates the expression of HR genes by sponging tumor-suppressive miR-421. **A,** Venn diagram displaying the miRNAs predicted to bind to *MALAT1* transcript using four miRNA prediction tools binding computational tools, namely LncBaseV.2, miRanda, NPInter v4.0, and miRTar. **B,** Mature miR-421 sequence and its seed sites within 3′UTR of *BRCA1, ATM* and *RAD51*. The seed sequence of miR-421 is shown in red while the target sequence is depicted in blue. **C,** Bar plot depicting the relative expression of miR-421 and *MALAT1* in 22RV1-miR-421 cells and control cells. **D,** qPCR depicting relative expression of HR genes in the same cells as in C. **E,** Quantitative PCR depicting relative expression of HR genes in 22RV1-shSCRM and -sh*MALAT1* cells transfected with nontargeting antagomiR or antagomiR-421. **F,** Schematic illustrating the predicted miR-421–binding sites near the 3′ end of *MALAT1*. **G,** Illustration of luciferase reporter construct with the wild-type or mutated (transformed residues in red) miR-421–binding sites on *MALAT1* 3′ end downstream of the firefly luciferase reporter gene. **H,** Bar plots depicting the luciferase reporter activity in HEK293T cells cotransfected with *MALAT1*-WT or *MALAT1*-mut construct with nontargeting mimics or miR-421 mimic. **I,** RIP followed by real-time qPCR analyses demonstrating enrichment of *MALAT1* and miR-421 with AGO2 antibody–bound beads in comparison to IgG (control antibody) in 22RV1 cells. The experiments were performed in triplicate with biologically independent samples (*n* = 3); the data represent mean ± SEM. The statistical analysis of differences was calculated using one-way ANOVA with Dunnett multiple-comparisons *posthoc* test for panels C–E and H–I.

Having established that *MALAT1* enhances the expression of HR genes, we next sought to explore the underlying mechanistic basis. To determine whether *MALAT1* directly binds to HR proteins and modulates their expression, we performed ChIRP assay (32), wherein we used biotinylated antisense oligonucleotides targeting *MALAT1* to retrieve the *MALAT1*-interacting proteins and examined the expression of critical DDR genes by immunoblotting. The enrichment of *MALAT1* was confirmed by qRT-PCR (Supplementary Fig. S5A), while the interaction with HR proteins, namely, BRCA1 and RAD51, was examined by immunoblotting. However, to our surprise, immunoblot data using *MALAT1*-biotinylated probes failed to show direct interaction with HR proteins, in contrast, a remarkable interaction between *MALAT1* and EZH2 was noted which was used as positive control (Supplementary Fig. S5B). Alternatively, we also cross-verified these results by performing RNA immunoprecipitation (RIP) using antibodies against BRCA1/2 and RAD51. Likewise, no interaction with BRCA1 or BRCA2 was observed in RIP assay, while a remarkable enrichment with the RAD51 antibody was noted, indicating that RAD51 directly interacts with *MALAT1* (Supplementary Fig. S5C). Nevertheless, RIP assays often exhibit nonspecific interactions that may form after cell lysis, thus RNA-centric methods such as ChIRP are preferred to determine the protein interactome of lncRNAs (45, 46). Hence, we concluded that *MALAT1* regulates the expression of HR proteins indirectly, either by modulating transcription factors or possibly by posttranscriptional regulation via miRNAs. Moreover, *MALAT1* has been reported to function as a competing endogenous RNA (ceRNA) or miRNA sponge that interacts with miRNAs and in turn, enhances the expression of downstream target genes (47). In tandem with this, several miRNAs have been shown to modulate the expression of genes associated with the HR pathway (48). Therefore, we investigated whether *MALAT1* could protect the proteins associated with the HR pathway by sponging the target miRNAs. To examine the miRNAs that might bind to the *MALAT1* transcript, we used four miRNA-binding prediction tools, namely LncBase v.2 (49), miRanda (50), NPinter v4.0 (51), and miRTar (52). About 45 miRNAs were identified in common by all four tools (Fig. 5A; Supplementary Table S4). Surprisingly, miR-338–5p and miR-421, which we previously validated as tumor suppressor miRNAs in prostate cancer (53), were also present among the predicted miRNAs that interacted with *MALAT1* (Fig. 5A). Moreover, miR-421 has been demonstrated to bind to the 3′ UTR of *ATM* and downregulate its expression in neuroblastoma, HeLa, and CRPC cells (54, 55). Besides, ectopic overexpression of miR-421 disrupts S-phase cell-cycle checkpoints and augments radiosensitivity (54). In addition, miRNA-binding prediction tools suggested that miR-421 can bind to several DDR genes, including *BRCA1*, *RAD51* and *ATM*, suggesting that miR-421 acts as a crucial player in the HR pathway (Fig. 5B). Intrigued by this observation, we generated stable miR-421–overexpressing 22RV1 cells (Fig. 5C) and examined the expression of *MALAT1* and major HR genes. Interestingly, overexpression of miR-421 in 22RV1 cells suppresses the expression of *MALAT1* (Fig. 5C) as well as key HR genes including *ATM, RAD51*, *BRCA1*, and *BRCA2* (Fig. 5D). Furthermore, to ascertain whether the decrease in expression of HR proteins in *MALAT1*-silenced cells is indeed due to miR-421–mediated posttranscriptional regulation, we transfected 22RV1-sh*MALAT1* and shSCRM cells with antagomiR (anti-miR) targeting miR-421. As expected, 22RV1-shSCRM transfected with anti-miR-421 showed a modest increase in the expression of HR genes while the *MALAT1*-depleted cells exhibited a robust increase, indicating that the decrease in the expression of HR genes upon *MALAT1* silencing could be mediated by miR-421 (Fig. 5E).

Furthermore, to examine the possible interaction between *MALAT1* and miR-421, we cloned the fragment of *MALAT1* harboring putative miR-421 (+6,501–6,708 bp; Fig. 5F) binding sites in a luciferase reporter construct (Fig. 5G). Interestingly, the luciferase activity of *MALAT1*-WT reporter constructs was significantly restrained upon cotransfection with respective miR-421 mimics compared with the control (miR-NT; Fig. 5H). Moreover, to confirm the specificity of the interaction, key residues within the predicted seed sequence were mutated and luciferase assay was performed. As expected, the mutant constructs failed to show any noticeable response upon miR-421 mimic transfection (Fig. 5H), indicating that *MALAT1* directly interacts with miR-421 via the predicted target site. Given that overexpression of miR-421 significantly suppresses the *MALAT1* expression (Fig. 5C) and a miR-421–binding site on the *MALAT1* transcript located adjacent to the AGO2-binding region (Fig. 5G), we speculate that miR-421 posttranscriptionally suppresses *MALAT1* expression via RNA-induced silencing complex. To confirm this, we performed RIP using an AGO2 antibody and noticed a robust enrichment of *MALAT1* as well as miR-421 with AGO2-bound beads in comparison with IgG (Fig. 5I). Collectively, these findings provide irrevocable evidence that a double-negative feedback loop between *MALAT1* and miR-421 modulates the expression of HR genes in prostate cancer.

### *MALAT1* Knockdown Sensitizes Prostate Cancer Cells to PARPi

**FIGURE 6:**
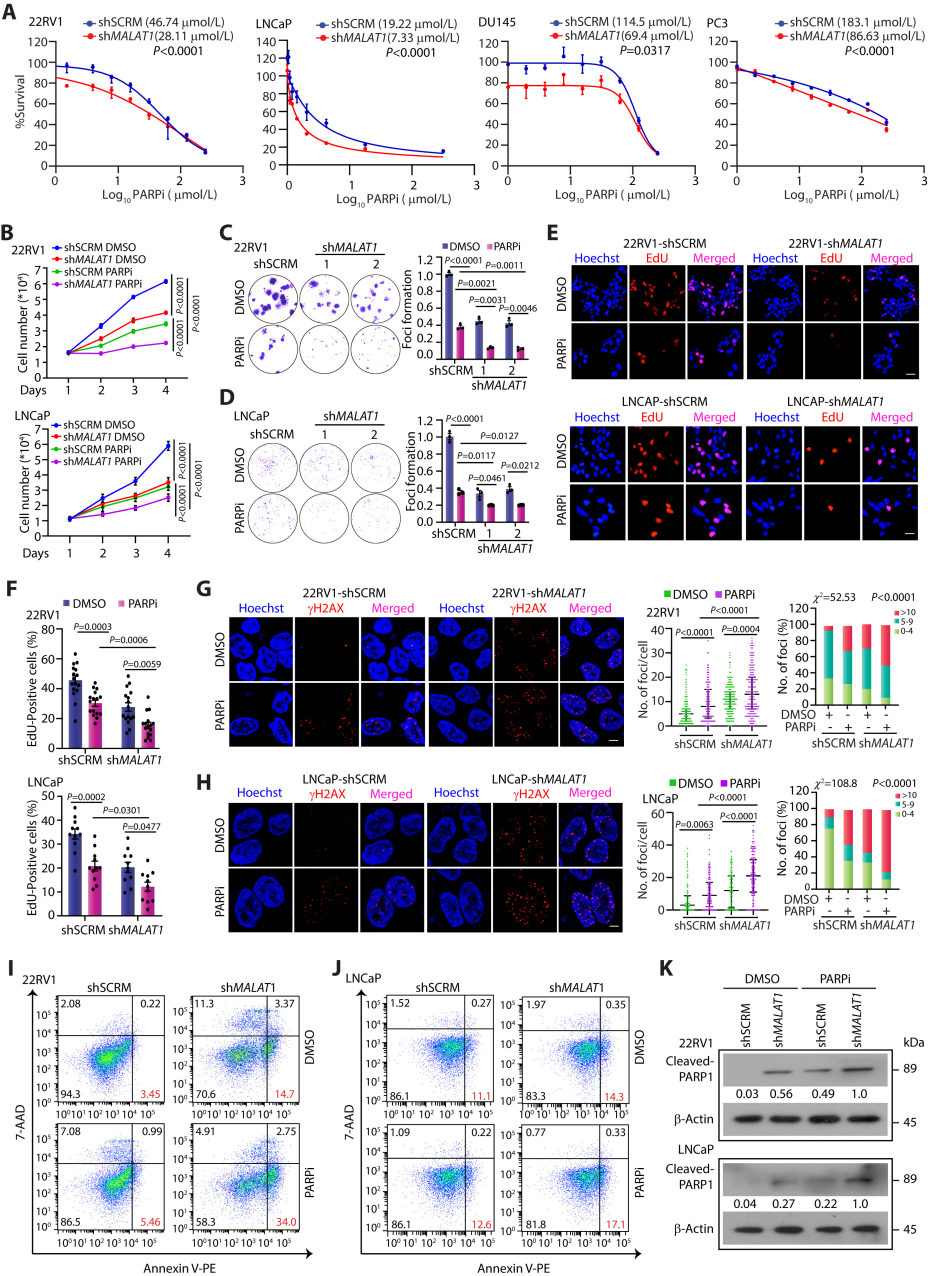
*MALAT1* depletion induces “BRCAness” and confers sensitivity to PARPi. **A,** Cell cytotoxicity assay for determining IC_50_ value of olaparib in SCRM control and *MALAT1*-silenced prostate cancer cells. The IC_50_ values were calculated by generating a dose–response curve using GraphPad Prism software. **B,** Line graph showing relative decrease in cell viability on olaparib treatment (10 µmol/L) in *MALAT1*-silenced 22RV1 and LNCaP cells as compared with scrambled control. The drug was replenished every 24 hours at the indicated time points. **C,** Foci formation assay in 22RV1-shSCRM and -sh*MALAT1* cells following treatment with olaparib (5 µmol/L) or vehicle control for 15 days. Inset showing representative images of foci. **D,** Foci formation assay in LNCaP-shSCRM and -sh*MALAT1* cells following treatment with olaparib (2 µmol/L) or vehicle control for 15 days. Inset showing representative images of foci. **E,** Representative confocal images for EdU uptake in *MALAT1*-deficient 22RV1 and LNCaP cells followed by olaparib (10 µmol/L) treatment for 48 hours. Scale bar, 50 µm. **F,** Bar graph showing quantification of EdU staining after 48-hour treatment with olaparib in the indicated cells. **G,** Representative confocal images for γH2AX foci (red) in the same cells as in C upon olaparib (10 µmol/L) treatment for 48 hours. The nucleus was visualized by Hoechst 33342 (blue). Scale bar, 10 µm. Quantification of the number of γH2AX-positive foci in the indicated cells. Bar plot showing the percentage of cells with the indicated number of foci/nuclei in the same cells. The *P* value for the χ^2^ test is indicated. **H,** Same as G, except LNCaP-shSCRM and LNCaP-sh*MALAT1* cells. **I,** Flow cytometry–based apoptosis assay using Annexin V-PE and 7-AAD staining in the same cells as in B upon olaparib (10 µmol/L) treatment for 48 hours. The percentage of the apoptotic cell population was calculated using FlowJo software. **J,** Same as I, except LNCaP-shSCRM and LNCaP-sh*MALAT1* cells. **K,** Immunoblot showing the change in expression of cleaved PARP in the same cells as in B upon olaparib (10 µmol/L) treatment for 48 hours. β-Actin was used as an internal control. The experiments were performed with *n* = 3 biologically independent samples; the data represents mean ± SEM. Extra sums of the square F-test was used to compute the statistical significance and compare the curves in A. The statistical analysis of difference was computed using two-way ANOVA with Tukey multiple-comparison *posthoc* test for B–D and F–H, while the *χ*^2^ test was used for G and H.

Cancer cells with a dysfunctional HR pathway heavily rely on PARP enzymes for removing damaged lesions and ensuring their survival. Any additional pharmacologic assault with PARPi or DNA-damaging drugs such as cisplatin, oxaliplatin, and carboplatin results in the accumulation of DNA lesions, eventually leading to cell death (56, 57). Because *MALAT1* knockdown contrives HR deficiency in prostate cancer, we speculated that *MALAT1-*deficient cells would be vulnerable to chemotherapeutic agents that target DNA repair. To examine this, *MALAT1-*silenced prostate cancer cells were treated with varying concentrations of olaparib, an FDA-approved PARPi, and analyzed for any change in drug response. Interestingly, *MALAT1* depletion in prostate cancer cells harboring mutations in *BRCA1/2*, namely 22RV1 and LNCaP showed a remarkable increase in drug sensitivity, while prostate cancer cells with no predicted biallelic mutations in canonical HR genes, that is, DU145 and PC3 cells (58), exhibited a modest increase in PARP sensitivity upon *MALAT1* knockdown (Fig. 6A). *MALAT1-*silenced prostate cancer cells exhibited decreased cell proliferation compared with shSCRM cells, while the effect was more pronounced (∼80%) in olaparib-treated cells (Fig. 6B), indicating that *MALAT1* depletion augments olaparib activity. In addition, the colony-forming ability of *MALAT1-*silenced prostate cancer cells was significantly impaired in the presence of olaparib in PARPi-sensitive (22RV1 and LNCaP) as well as PARPi-insensitive (DU145 and PC3) prostate cancer cell lines (Fig. 6C and D; Supplementary Fig. S6), suggesting that *MALAT1* depletion could sensitize HR-deficient as well as HR-proficient prostate cancer cells to olaparib**.** As *MALAT1* depletion markedly enhances cellular sensitivity to PARPi, we examined the effect of olaparib treatment on cell-cycle profiles in *MALAT1-*silenced prostate cancer cells by performing EdU staining. *MALAT1-*deficient prostate cancer cells upon olaparib treatment show a decrease in the S-phase population compared with the shSCRM control (Fig. 6E and F).

To assess whether *MALAT1* depletion impairs the DNA repair system and sensitizes prostate cancer to olaparib, we examined the frequency of γH2AX foci in olaparib-treated *MALAT1-*silenced cells. Almost 2-fold higher γH2AX foci per cell were noted in olaparib-treated *MALAT1-*silenced cells compared with shSCRM control (Fig. 6G and H), suggesting that *MALAT1* deficiency exacerbates olaparib-induced DNA damage in prostate cancer cells. Because the accumulation of DNA lesions instigates apoptosis, olaparib-treated sh*MALAT1* and shSCRM prostate cancer cells were further examined for cell death by AnnexinV-7AAD staining. A marginal increase in the early apoptotic cell population was observed in *MALAT1-*ablated prostate cancer cells compared with their respective shSCRM controls, while upon olaparib treatment the number of apoptotic cells was markedly increased (Fig. 6I and J). Consistent with this, the level of cleaved PARP was also increased in olaparib-treated sh*MALAT1* cells compared with shSCRM control (Fig. 6K). Furthermore, genetic depletion of PARP1 in HR-proficient as well as HR-deficient prostate cancer cells suppresses the expression of DDR genes in both, shSCRM as well as sh*MALAT1* cells, nevertheless, the sh*MALAT1* cells exhibit a more notable reduction in expression, suggesting that PARP1 depletion cooperatively suppress the expression of HR genes in *MALAT1*-silenced cells (Supplementary Fig. S7). Collectively, our results provide compelling evidence that *MALAT1* depletion enhances the sensitivity to PARPi; hence, targeting both simultaneously will be a promising therapeutic approach for patients with advanced stage prostate cancer, who often develop resistance to conventional therapeutic strategies.

### *MALAT1* Ablation Enhances the Sensitivity of Prostate Cancer to PARPi

**FIGURE 7:**
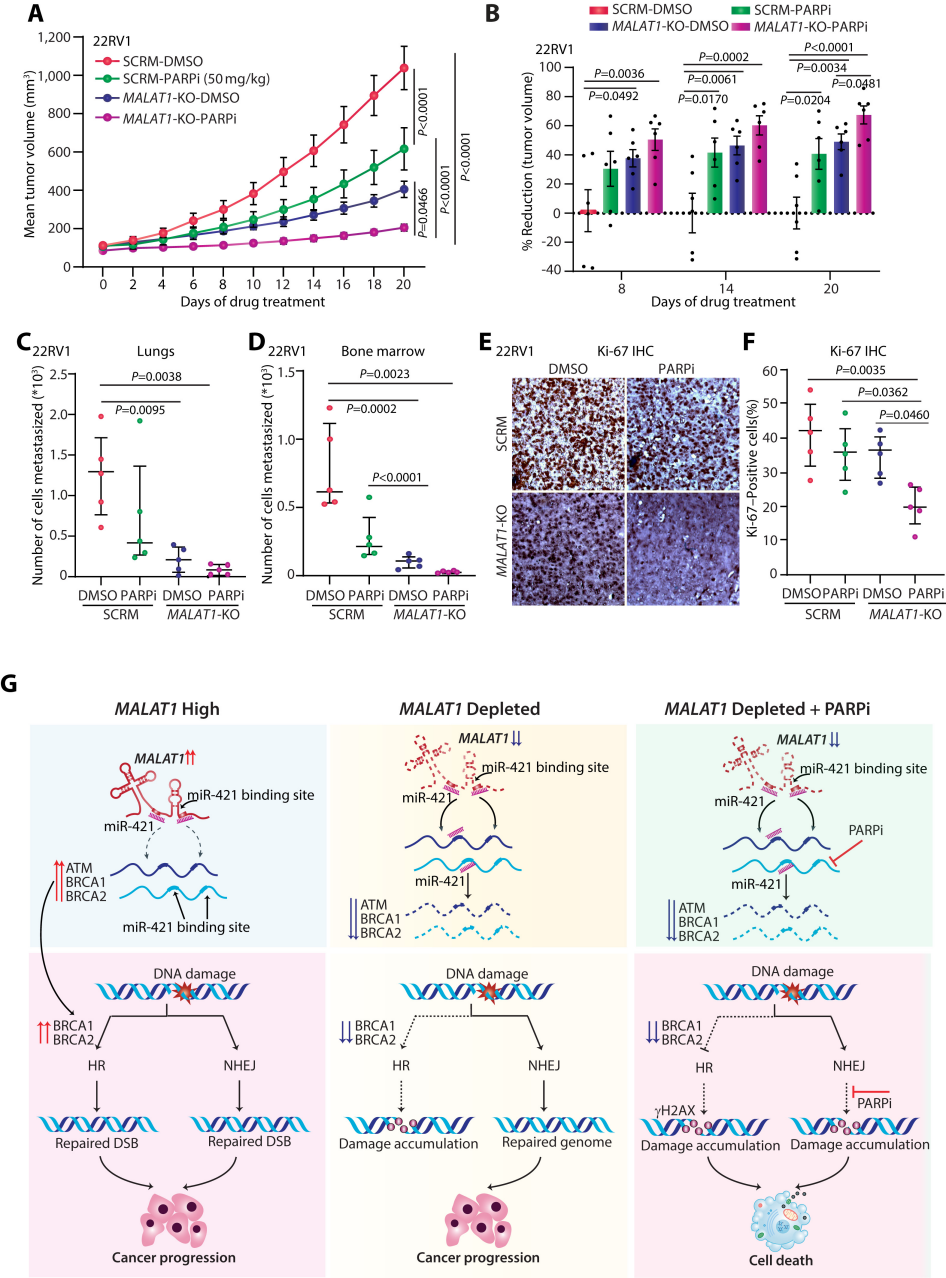
*MALAT1* ablation confers sensitivity to PARPi. **A,** Mean tumor volume of xenografts generated by implanting 22RV1-SCRM and 22RV1-*MALAT1* KO cells in NOD-SCID mice and randomized into two groups (*n* = 6 each), namely, vehicle control and olaparib (50 mg/kg). **B,** Bar plot showing percent tumor reduction in the same mice as mentioned in A. **C,** Scatter dot plot showing the number of cells metastasized to the lungs in xenografted mice as mentioned in A. **D,** Same as C, except cells metastasized to bone marrow. **E,** Representative images depicting IHC staining for Ki-67 in formalin-fixed paraffin-embedded tumor xenograft specimens as in A. **F,** Scatter dot plot showing quantification of Ki-67 expression in the tumor tissue sections of the mice xenografts as in A. **G,** Schema depicting that *MALAT1* inhibition perturbs HR machinery via miR-421 and this in turn enhances the sensitivity toward PARPi. *MALAT1* sponges miR-421 which in turn enhances the DNA repair activity of prostate cancer cells and enables them to proliferate and survive even in the presence of therapy-induced damage. While *MALAT1* depletion disrupts the HR machinery, the NHEJ pathway takes over and repairs the damage, and aids in the survival of prostate cancer cells. However, when, *MALAT1*-depleted cells are treated with pharmacologic inhibitors of PARP1, a type of enzyme that helps to repair damaged DNA via the NHEJ pathway, the cells are unable to repair the breaks brought on by treatment with PARP1 inhibitors and ultimately result in cell death. For A and B, the data represent mean ± SEM and the statistical difference was computed using two-way ANOVA with Tukey multiple comparison test while for C, D and F, the data are presented as median (middle line) with interquartile range and one-way ANOVA with Tukey multiple comparison test was applied.

On the basis of our *in vitro* results and previous research emphasizing the role of *MALAT1* in prostate tumorigenesis and metastasis, we next examined the impact of PARPi in *MALAT1*-high versus low prostate cancer tumors. For this, we implanted 22RV1-SCRM and 22RV1-*MALAT1* knockout cells subcutaneously in athymic immunodeficient mice (*n* = 12 per group). The mice were randomly divided into two groups (*n* = 6) once the tumors reached a palpable size (average ∼100 mm^3^), the intended vehicle control and PARPi (50 mg/kg) were administered. A significant decrease in tumor volume was noted after treatment with PARPi in both 22RV1-SCRM and 22RV1 *MALAT1* KO groups; however, the percentage reduction in tumor volume was more pronounced in the *MALAT1-*KO group (∼80%) compared with the 22RV1-SCRM (∼55%) at day 20 upon treatment with PARPi (Fig. 7A and B). This observation corroborates with our *in vitro* data, wherein the anticancer properties of PARPi were enhanced upon *MALAT1* knockdown in prostate cancer cells. Subsequently, to examine the coinhibitory potential of *MALAT1* and PARP1 in distant tumor metastasis, we determined the levels of human-specific *Alu* sequences in the lungs and bone marrow collected from xenografted mice. Mice implanted with *MALAT1* KO cells showed a significant reduction in lung and bone metastases upon PARPi treatment, as compared with SCRM control cells (Fig. 7C and D). On the other hand, mice implanted with 22RV1 SCRM cells showed a significant reduction in bone metastasis (Fig. 7D) upon PARPi treatment while no significant change was observed in lung metastasis when compared with vehicle control (Fig. 7C). Furthermore, to investigate the effect of coinhibition of *MALAT1* and PARP in tumor xenografts, we performed IHC staining for the cell proliferative marker Ki-67. As speculated, a significant decrease in Ki-67 levels was observed in mice implanted with *MALAT1* KO cells upon PARPi treatment, as compared with SCRM control cells (Fig. 7E and F). Conclusively, our mice xenograft studies showed that olaparib-mediated reduction of prostate tumor growth and metastases is potentiated by inhibition of *MALAT1.* These findings suggest that targeting *MALAT1* enhances the vulnerability of prostate cancer to PARPi therapy.

## Discussion

Advanced stage prostate cancer is characterized by higher metastatic potential, enhanced biochemical recurrence, and poor patient survival (59). Identification of molecular mechanisms orchestrating the progression to CRPC will facilitate development of novel targeted therapeutic strategies for the disease. In this study, we used an integrated bioinformatics approach to identify the molecular factor(s) associated with mCRPC and discovered *MALAT1*, an oncogenic lncRNA, is significantly upregulated in patients with mCRPC compared with localized cases. Consistent with this, previous studies also demonstrated frequent upregulation of *MALAT1* in advanced stage prostate cancer which positively associates with an aggressive clinical phenotype (60, 61). Furthermore, *MALAT1* upregulation has also been linked to poor prognosis in several malignancies (35, 37), suggesting that it may be associated with drug resistance.

Here, for the first time, we demonstrate that *MALAT1* is essential for preserving the genomic integrity in advanced stage prostate cancer and protects tumor cells from the damage caused by anticancer agents by enhancing DNA repair pathways, which in turn induces resistance to chemotherapeutic drugs and facilitates the survival of cancer cells. Mechanistically, we show that targeting *MALAT1* induces DSBs and apoptosis, which makes prostate cancer cells vulnerable to DNA repair inhibitors, such as olaparib, and DNA-damaging agents like doxorubicin. Contrary to the well-documented inactivating mutations in the HR pathway, herein we found that the core HR genes are upregulated in patients with CRPC and positively correlate with *MALAT1* expression. In accord with this, several recent reports have also demonstrated that HR genes are frequently upregulated in lethal and advanced stage prostate cancer tumors, that is, CRPC (9) and neuroendocrine prostate cancer (6, 9). Furthermore, HR proteins are known to be elevated in several other therapy-resistant malignancies such as glioblastoma (11) and triple-negative breast cancer (62); however, the underlying biological mechanism(s) involved in their upregulation remain unknown and require extensive molecular characterization.

In this study, we found that *MALAT1* modulates the expression of key HR proteins, namely *BRCA1*/2 and *RAD51*, but does not interact with them directly. Likewise, Huang and colleagues showed that *MALAT1* indirectly modulates the expression of BRCA1 in non–small cell lung carcinoma (63). To delineate the possible connection between *MALAT1* and HR genes, we explored the possibility of posttranscriptional regulation via miRNAs and found that *MALAT1* sequesters miR-421, a tumor suppressor miRNA that regulates the expression of HR genes. In addition, our findings also demonstrate the presence of a double-negative feedback loop between *MALAT1* and miR-421; wherein *MALAT1* sequesters miR-421 and averts its binding to target proteins, while miR-421 posttranscriptionally suppresses *MALAT1*, suggesting that reciprocal regulation between *MALAT1* and miR-421 regulates the expression of HR genes in prostate cancer. Nevertheless, further in-depth investigation is needed as we speculate that transcriptional and epigenetic alterations induced by silencing *MALAT1*, might also modulate the expression of HR proteins.

Collectively, our findings demonstrate that the HR deficiency induced by *MALAT1* depletion phenocopies “BRCAness” and augments sensitivity to clinically approved DNA repair inhibitors such as olaparib (Fig. 7G). In consonance with our results, several recent reports also demonstrate that *MALAT1* depletion enhances sensitivity toward chemotherapeutic drugs like docetaxel (64), oxaliplatin (65), cisplatin (66), and cytarabine (67) by sponging miR-200b, miR-324–3p, miR-145, and miR-96, respectively. *MALAT1* also increases the transcriptional activity of YAP1, which can alter the HR as well as the non–homologous end joining (NHEJ) pathway, to confer radioresistance to colorectal cancer cells (68). Furthermore, *MALAT1* has been shown to modulate the alternative NHEJ (A-NHEJ) pathway by directly interacting with LIG3 and PARP1 in multiple myeloma cells and targeting *MALAT1* enhances sensitivity to PARPi or proteasomal inhibitors (69). These findings, in line with ours, allude to the fact that *MALAT1* is a crucial molecular modulator of the DDR pathway that may augment chemoresistance in advanced stage cancer.

PARPi, such as olaparib and rucaparib have shown promising results in the clinical management of multiple malignancies, including mCRPC, particularly in patients harboring mutations in the HR genes (70). However, the proportion of patients with prostate cancer harboring HR mutations is less than 20%; hence, targeting alternative genetic and epigenetic factors that selectively regulate the HR pathway may sensitize HR-proficient prostate cancer to PARPi. Moreover, several preclinical studies have indicated that androgen receptor (AR) signaling antagonists may enhance the sensitivity of prostate cancer cells to PARPi. Specifically, AR antagonists demonstrate contextual synthetic lethality with PARPi by modulating the expression of key HR proteins such as BRCA1/2 and RAD51, and potentially enhance the vulnerability of prostate cancer cells on alternative DNA repair mechanisms, such as PARP-mediated repair (71). Considering this, the combination of enzalutamide and olaparib in patients with mCRPC, particularly those who have alterations in DNA repair genes was assessed in Profound clinical trial (NCT02987543). The results showed that patients who received the combination therapy had better overall, and radiographic progression-free survival (rPFS) compared with those receiving enzalutamide alone (72). Furthermore, recently the FDA-approved talazoparib in combination with enzalutamide for the treatment of mCRPC patients harboring HR gene mutations, which markedly improved the rPFS in comparison with placebo and enzalutamide in TALAPRO-2 clinical trial (73). In addition, recent reports also suggest that *MALAT1* expression can be modulated by androgens, while it reciprocally enhances the transcriptional activity of AR by acting as its coactivator (74). However, the specific interactions and mechanisms are still under investigation, and we conjecture that there is a direct connection between *MALAT1*, AR signaling, and the HR pathway.

Furthermore, multiple lines of evidence show that small-molecule inhibitors against bromodomain and extra-terminal motif (BET) proteins (75), PI3K (PI3K/AKT; ref. 76), and histone deacetylases (HDAC; ref. 77) synergize with PARPi by suppressing the expression of BRCA1 and RAD51 while CDK1 inhibitors disrupt the recruitment of BRCA1 to DNA damage sites and in turn, enhance sensitivity to PARPi (78). Pharmacologic inhibition of DNA methyltransferases (DNMT) increases PARPi sensitivity by enhancing PARP1-DNA binding (79). Our data suggests the presence of a double-negative feedback loop between *MALAT1* and PARP1 in both AR-negative and AR-positive prostate cancer cell lines (Supplementary Fig. S7). It has been shown that PARPi traps both PARP1 and PARP2 at the damaged DNA, where PARP–DNA complexes show more cytotoxicity than unrepaired SSBs caused by PARP inactivation (80). Recently, a competitive binding study showed that PAR binding releases PARP1 from DNA via its WGR domain (Trp-Gly-Arg rich), and PAR induces catalytic stimulation of PARP1, while impeding the DNA-dependent stimulation. This report stresses on the role of high-affinity PAR reader domains of PARP1 and proposes a novel mechanism of allosteric regulation of DNA-dependent and DNA-independent activities of PARP1 (81). Thus, considering these lines of evidence, we speculate that PARPi might be affecting other key enzymes involved in DNA repair in addition to PARP1.

On the basis of these preclinical studies, several drug combination approaches are being evaluated in clinical trials, for instance, the clinical trial TRAP (NCT03787680) is evaluating the efficacy and safety of olaparib in combination with an ATR inhibitor (AZD6738), while another clinical trial, NCT02893917 is investigating the efficacy of a combinatorial regimen including cediranib, a VEGF receptor inhibitor, and olaparib for the treatment of patients with mCRPC (82). Along similar lines, our findings provide a compelling rationale for conducting clinical trials in patients with advanced stage disease to investigate the safety and efficacy of combinatorial therapy using *MALAT1* antisense oligonucleotides/GAPmers or small-molecule inhibitors against *MALAT1* with PARPi or DNA-damaging agents like cisplatin. In addition, this study provides an important molecular connection between *MALAT1*, miR-421, and HR pathway in prostate cancer. Understanding the precise involvement of miR-421 in the HR pathway may contribute to the development of miR-421 as a DNA damage response signature and a possible therapeutic target for patients with prostate cancer. Conclusively, our findings indicate that oncogenic lncRNA *MALAT1* protects prostate cancer tumor cells from anticancer agents by initiating the HR pathway, revealing a potential therapeutic vulnerability that can be exploited by targeting *MALAT1* or overexpressing miR-421 in conjunction with PARPi.

## Supplementary Material

Supplementary Methods Supplementary Methods

Supplementary Figure S1 MALAT1 is upregulated in prostate cancer and positively associates with aggressive clinical phenotype.

Supplementary Figure S2 MALAT1 positively associates with mesenchymal and stemness markers in prostate cancer patients.

Supplementary Figure S3 MALAT1 regulates the DNA repair pathway in prostate cancer.

Supplementary Figure S4 MALAT1 depletion restrains cell cycle progression in prostate cancer.

Supplementary Figure S5 MALAT1 does not interact with HR proteins.

Supplementary Figure S6 MALAT1 depletion enhances sensitivity to PARP inhibitors in HR proficient PCa cells.

Supplementary Figure S7 MALAT1 and PARP depletion cooperatively suppress expression of HR genes in HR-deficient as well as HR-proficient PCa cells.

Supplementary Table S1 Supplementary Table S1: List of the Primers

Supplementary Table S2 Supplementary Table S2: Genes upregulated in metastatic prostate cancer patients in comparison to localized cases.

Supplementary Table S3 Supplementary Table S3: Biological pathways downregulated on MALAT1 ablation, predicted by DAVID analysis.

Supplementary Table S4 Supplementary Table S4: Computational prediction of microRNAs with putative binding sites on the MALAT1transcript.
