# Supplementary material for: Targeting MALAT1 Augments Sensitivity to PARP Inhibition by Impairing Homologous Recombination in Prostate Cancer
Source: Cancer Res Commun. 2023 Oct 9;3(10):2044–61. doi: 10.1158/2767-9764.CRC-23-0089 (PMC10561629; doi:10.1158/2767-9764.CRC-23-0089)
Supplement: Supplementary Methods [file crc-23-0089-s01.pdf]

Supplementary data

Targeting *MALAT1* Augments Sensitivity to PARP Inhibition by Impairing Homologous Recombination in Prostate Cancer

Anjali Yadav[1], Tanay Biswas[1], Ayush Praveen[1], Promit Ganguly[1], Ankita Bhattacharyya[1], Ayushi Verma[2], Dipak Datta[2] and Bushra Ateeq[1,3,4]*

[1]Molecular Oncology Laboratory, Department of Biological Sciences and Bioengineering, Indian Institute of Technology Kanpur, Kanpur, U.P., 208016, India.

[2]Division of Cancer Biology, CSIR-Central Drug Research Institute, Lucknow, U.P., 226031, India.

[3]Mehta Family Centre for Engineering in Medicine, Indian Institute of Technology Kanpur, Kanpur, U.P., 208016, India.

[4]Centre of Excellence for Cancer - Gangwal School of Medical Sciences and Technology, Indian Institute of Technology Kanpur, Kanpur, U.P., 208016, India.

Running Title: *MALAT1* silencing enhances sensitivity to PARP inhibitors.

Conflict of interest: The authors declare no potential conflicts of interest.

***Corresponding Author and Lead Contact**:
Dr. Bushra Ateeq,
Molecular Oncology Laboratory,
Department of Biological Sciences and Bioengineering,
Mehta Family Centre for Engineering in Medicine,
Indian Institute of Technology Kanpur,
Kanpur, 208016, India
Phone: +91 512 2594083
Fax: +91 512 2594010
Email: bushra@iitk.ac.in

Supplementary material and methods:

Real-time quantitative PCR: Total RNA was extracted using TRIzol (Ambion) and reverse transcribed using the first strand cDNA synthesis kit (Genetix Biotech Asia Pvt. Ltd) as recommended by the manufacturer. The expression of the target gene was confirmed by performing qPCR using SYBR Green PCR master-mix (Applied Biosystems) and primers mentioned in **Supplementary Table S1**. *GAPDH* was used as an internal reference, and the relative expression of the target gene was calculated using the $-\Delta\Delta Ct$ method.

Immunoblotting: The cells were lysed in RIPA buffer supplemented with phosphatase (Calbiochem) and protease (VWR) inhibitor. The BCA assay kit was used to quantify the isolated protein samples (GE Healthcare). The protein samples were heat-denatured and resolved on SDS-PAGE. The resolved proteins were transferred to a PVDF membrane (GE Healthcare) and blocked with 5% nonfat dry milk for 1 hour at room temperature. The membrane was incubated with the following concentrations of primary antibodies overnight at 4°C. An antibody dilution of 1:1000 was used for N-cadherin (Abcam, ab98952, RRID:AB_10696943), E2F1 rabbit (CST, 3742S, RRID:AB_2096936), cleaved PARP (CST, 9541, RRID:AB_331426), BCl2 (CST, 2870, RRID:AB_2290370), Bcl-xL (CST, 2764, RRID:AB_2228008); 1:2000 dilution was used for RAD51 (Abcam, ab133534, RRID:AB_2722613), 1:5000 dilution was used for E-cadherin (CST, 3195, RRID:AB_2291471) and β-actin (Abcam, ab6276, RRID:AB_2223210). DNA Damage Antibody Sampler Kit (CST, 9947, RRID:AB_836871) was used to examine the expression of BRCA1, γ-H2AX and pCHEK2. The membrane was then incubated for two hours at room temperature with a 1:10000 dilution of horseradish peroxidase (HRP) conjugated secondary anti-mouse antibody (Jackson ImmunoResearch Laboratories, Cat # 115-035-003, RRID:AB_10015289) or anti-rabbit antibody (Cat # 111-035-144, RRID:AB_2307391). After

this, the signals were detected through an enhanced chemiluminescence system (Thermofisher) as directed by the manufacturer.

Cell migration assay: The cell migration assay was performed in a Transwell Boyden chamber with an 8μm pore size (Corning) as described before (1). The upper compartment was seeded with $1*10^5$ cells suspended in serum-free culture medium, while the lower compartment was filled with conditioned media supplemented with 20% fetal bovine serum (FBS). After 24-30 hours, the migrated cells were fixed using 4% paraformaldehyde (PFA) in 1X phosphate buffered saline (PBS) and stained with crystal violet (0.5% w/v). An upright Nikon microscope was used to examine and photograph migrating cells. Further, for quantification, the cells were de-stained with 10% v/v glacial acetic acid (GAA) and absorbance was measured at 550nm.

Prostatosphere assay: 10,000 cells per condition were plated in a serum-free stem cell medium consisting of DMEM-F12 (1:1, Invitrogen), 1X B27 (Invitrogen), 20ng/ml EGF (Invitrogen), and 20ng/ml FGF (Invitrogen, ref. 2, 3). Every 3rd day, the prostatospheres formed were disassociated into single-cells and then re-plated in fresh medium for multiple generations. The experiment was terminated after 12 days, and bright-field images of the spheres were captured using an Axio Observer Z1 inverted fluorescence microscope (Carl Zeiss) equipped with an apotome device. Further, the prostatospheres formed in all the groups were calculated and analyzed for sphere- forming efficiency using ImageJ software (RRID:SCR_003070). The spheroids were also subjected to RNA isolation and analyzed for expression of stemness genes.

Immunofluorescence staining: The cells were plated at 60% confluency and incubated for 36 hours. The cells were washed twice with PBS and then fixed with 4% PFA in PBS. The fixed cells were washed thrice with PBS and then permeabilized with 0.3% Triton X-100 in PBS (PBS-T). The cells were blocked with 5% normal goat serum (NGS) in PBS-T for 2 hours at room

temperature and then incubated with the following primary antibodies: CD117 (1:400, CST, 3308, RRID:AB_659956), CD44 (1:400, CST, 3570, RRID:AB_2076465), and γ-H2AX (1:100, CST, 2577, RRID:AB_2118010), γ-H2AX (1:100, Santa Cruz, sc517348, RRID:AB_2783871), BRCA1 (1:200, Santa Cruz, sc6954, RRID:AB_626761), RAD51 (1.1000, Abcam, ab133534, RRID:AB_2722613) at 4 °C overnight. Cells were washed thrice with PBS-T before being stained with DAPI (Sigma-Aldrich). The coverslips were then adhered to glass slides using an anti-fade Vectashield mounting medium (Vector Laboratories). Images were captured with a Carl Zeiss Axio Observer Z1 inverted fluorescence microscope fitted with an apotome device.

Immunophenotyping: Cells were grown to 80% confluence and dissociated using StemPro™ Accutase™ (ThermoFisher). The cells were re-suspended in PBS with 5% FBS at 10^6 cells/mL. Further, 100µl of cell suspension was incubated with CD117-APC (Miltenyi Biotec, 130-098-207, RRID:AB_2660103, 1:50), CD133-PE (Miltenyi Biotec, 130-113-670, RRID:AB_2726212, 1:50), and CD338-PE (Miltenyi Biotec, 130-105-010, RRID:AB_2657630, 1:50) for 1h on ice, then washed thrice with 1X-PBS supplemented with 5% FBS (4). The events were captured on BD Beckman Coulter's CytoFLEX platform and analyzed with FlowJo version 10.7 (RRID:SCR_008520).

Cell cycle distribution: For cell cycle distribution analysis, the cells were seeded at 50-60% confluency and grown for 48 hours to obtain cultures in the logarithmic growth phase. Further, for synchronization, the cells were growth arrested by serum deprivation for 12 hours. After this, the cells were trypsinized, and 10^6 cells per sample were re-suspended in 300µl PBS. For fixation, 0.7 ml of 100% ice-cold ethanol was added drop-by-drop with vortexing. The cells were placed on ice for 30 minutes. Following this, the fixed cells were stained with 50µg/ml propidium iodide (PI) (BioLegend, Cat#421301) according to the manufacturer's protocol. The acquired events were

analyzed using FlowJo software version 10.7's built-in univariate model to assess cell cycle distribution.

Apoptosis assay: *MALAT1*-silenced cells were stained with PE Annexin V and 7-Aminoactinomycin D (7AAD, BD Biosciences, Cat#559763) as recommended by the manufacturer's protocol. Quadrants were gated using the unstained, GFP, Annexin V (PE), and 7AAD (PerCP) single stained cells as controls and the cell were divided into four quadrants: the lower left quadrant represents the $Annexin^{-}$ and $7AAD^{-}$ population i.e. viable cells, the lower right quadrant represents $Annexin^{+}$ and $7AAD^{-}$ population i.e. early apoptotic, upper left quadrant represents $Annexin^{-}$ and $7AAD^{+}$ i.e. the necrotic) cells while the upper right quadrant represents $Annexin^{+}/7AAD^{+}$, i.e. the late apoptotic cells (5). Further, flowJo software v10.7 was used to analyze the data acquired on the BD FACS Melody cell sorter for each condition.

Chemosensitivity assay: To determine the IC_{50} values of the drugs, 2000 cells/well were seeded in the 96-well plates and treated with varying concentrations of Doxorubicin and 5-Fluorouracil, for 48 h. Likewise, IC_{50} values of the Olaparib in different PCa cells was determined by treating the cells with varying concentrations the drug for 96 h, wherein the drug was replenished after every 24 hours. Resazurin (Cayman Chemicals) was added to each well according to the manufacturer's instructions, followed by fluorescence measurement with emission-excitation at 590-530nm after 3.5 hours. The IC_{50} values of the drugs were calculated using linear approximation regression of the percentage survival versus the drug concentration.

EdU labeling assay: *MALAT1*-silenced PCa cells were stained with the Click-iT EdU Cell Proliferation Kit for Imaging (Thermofisher, C10338) as directed by the manufacturer's protocol. In brief, 25,000 cells were plated on glass coverslips in 12-well plates and incubated for 36 hours. Further, for synchronization, the cells were serum starved for 12 hours, before being treated with

10μM EdU for 2 hours at 37 ºC. Following that, the cells were fixed with 4% PFA and permeabilized with 0.3% PBS-T for 10 minutes. The fixed cells were stained with EdU Azide Alexa Fluor 555 for 30 minutes, and later the nucleus was stained with Hoechst 33342/DAPI for 20 minutes (Thermofisher, 62249). Vectashield mounting medium was used to adhere the coverslips to the glass slides (Vector Laboratories). Images were taken with a Carl Zeiss Axio Observer Z1 inverted fluorescence microscope fitted with an Apotome device. ImageJ software was used for the post-processing and quantification of the acquired images.

Cell proliferation assay: The cell proliferation assay was performed by plating 10,000 cells per well in a 12-well plate and counting the cells at the indicated time points. Cells were treated with 10μM Olaparib as well as DMSO control and cultured till the indicated time points. Cells were trypsinized at the designated time and counted using a hemocytometer.

Foci formation assay: *MALAT1*-depleted PCa cells (2000 cells/well) were cultured in serum-deprived conditions in RPMI-1640 medium (Gibco). The medium was replenished every 48 h with Olaparib (5μM for 22RV1, 2μM for LNCaP, 10μM for PC3 and DU145) along with DMSO control. After 2 weeks, the foci formed in each condition were fixed with 4% paraformaldehyde (PFA) and stained with crystal violet (0.1% w/v). After capturing images, the foci were destained using 10% v/v glacial acetic acid (GAA), and the absorbance was measured at 550 nm.

Generating stable miR-421 overexpression cell lines: Pre-miR-421 (miRBase accession ID: MIMAT0003339) was amplified from human genomic DNA. The amplified fragment and the lentiviral miRNA-expression vector (pLemiR) with turbo RFP (Open Biosystems) were then digested with *XhoI/NotI* restriction enzymes. The digested fragments were subsequently ligated and screened by colony PCR. Further, Sanger sequencing was used to confirm positive clones. To establish stable miR-421 overexpressing and vector control cells, 22RV1 cells were transfected

with 2μg of miR-421 pLemiR and an empty pLemiR construct using FuGENE HD transfection reagent (Promega). The transfection complex was replaced with fresh medium after 12 hours. The transfected cells were selected using 1μg/ml puromycin, 48 hours' post-transfection. To acquire single clones overexpressing these miR-421, single cells from the pooled population were injected into each well of a 96-well plate and cultured for a further 2-3 weeks under puromycin selection. Finally, quantitative PCR was used to examine single clones for miR-421 overexpression.

MicroRNA-*MALAT1* luciferase reporter assay: The genomic region of *MALAT1* harboring the miR-421 binding site (250 bp) was cloned into the pEZX-MT01 Firefly/Renilla dual-luciferase reporter vector (GeneCopoeia). Cells were grown at ~40-50% confluence in a 4-well plate and co- transfected with 30 pmol of miR-421 mimic and 25 ng of pEZX-MT01 constructs using lipofectamine RNAiMax (Invitrogen, ref. 6). Cells were harvested 24 hours after the second transfection using the lysis buffer supplied with the Dual-Glo Luciferase Assay Kit (Promega). The GloMax® 96 Microplate Luminometer (Promega) was used to measure the Firefly and Renilla luciferase activities according to the manufacturer's procedure. Renilla luciferase activity was considered as the normalization control.

AntagomiR (anti-miR) transfection: 22RV1-shSCRM and –sh*MALAT1* cells were seeded at 40- 50% confluency in a 6-well dish. After 36 h, 30 pmol of antagomiR targettingmiR-421 and non- targeting control (Qiagen) were transfected using lipofectamine RNAiMAX transfection reagent (ThermoFisher Scientific). Cells were transfected again after 24 h, and 48 h post-transfection the cells were harvested in TRIzol for RNA isolation.

RNA Immunoprecipitation (RIP): 22RV1 cells were seeded at ~80% confluency, after 36 h cells were scraped in 1X phosphate buffer saline (PBS) supplemented with 1X PIC. The lysate was centrifuged, and the pellet was re-suspended in RIP buffer consisting of Tris-HCl (50 mM, pH 7.9), sodium chloride (0.25 M,) Nonidet P-40 (1%, NP-40), EDTA (10 mM), 1X PIC and RNase inhibitor and incubated on ice for 30 min. Further, cell lysate was collected by centrifugation

at 12,000 rpm for 10 min and was incubated overnight with 4 μg of AGO2 (Abcam, ab32381, RRID:AB_867543), BRCA1 (CST, 9010, RRID:AB_2228244), RAD51 (Abcam, ab133534) and BRCA2 (Invitrogen, 234403) antibody or IgG at 4°C. Concurrently, protein-coated Dynabeads were pre-absorbed with 100 μg/ml BSA. The pre-absorbed beads were washed thrice with NT2 buffer consisting of Tris-HCl (50 mM, pH 7.4), sodium chloride (300 mM), magnesium chloride (1 mM), NP40 (0.05%), 1X PIC, and RNase inhibitor. The washed beads were then incubated with RNA-antibody adducts to form RNA- antibody-bead precipitates (7). The beads were washed three times with NT2 buffer before being digested with DNase I for 15 min at 37°C. Trizol was used to extract co-purified RNA, and Puregene's SuperScript kit was used to synthesize cDNA.

REFERENCES

1. Tiwari R, Pandey SK, Goel S, Bhatia V, Shukla S, *et. al.* SPINK1 promotes colorectal cancer progression by downregulating Metallothioneins expression. *Oncogenesis* 2015;4:e162.

2. Johnson S, Chen H, Lo P-K. In vitro tumorsphere formation assays. *Bio-protocol* 2013;3:3.

3. Dontu G, Abdallah WM, Foley JM, Jackson KW, Clarke MF, *et. al.* In vitro propagation and transcriptional profiling of human mammary stem/progenitor cells. Genes & development 2003;17:1253-70.

4. Tiwari R, Manzar N, Bhatia V, Yadav A, Nengroo MA *et. al*. Androgen deprivation upregulates SPINK1 expression and potentiates cellular plasticity in prostate cancer. *Nat Commun.* 2020;11:384

5. Goel S, Bhatia V, Kundu S, Biswas T, Carskadon S, *et. al*. Transcriptional network involving ERG and AR orchestrates Distal-less homeobox-1 mediated prostate cancer progression. *Nat. Commun.* 2021;12:5325-4247.

6. Bhatia V, Yadav A, Tiwari R, Nigam S, Goel S, *et. al.* Epigenetic silencing of miRNA-338-5p and miRNA-421 drives SPINK1-positive prostate cancer. *Clin. Cancer Res*. 2019;25:2755-2768.

7. Gagliardi M. and Matarazzo M.R. RIP: RNA Immunoprecipitation. *Methods Mol Biol*. 2016,1480, 73-86.
