# Supplementary material for: Targeting MALAT1 Augments Sensitivity to PARP Inhibition by Impairing Homologous Recombination in Prostate Cancer
Source: Cancer Res Commun. 2023 Oct 9;3(10):2044–61. doi: 10.1158/2767-9764.CRC-23-0089 (PMC10561629; doi:10.1158/2767-9764.CRC-23-0089)
Supplement: Supplementary Figure S1 — MALAT1 is upregulated in prostate cancer and positively associates with aggressive clinical phenotype. [file crc-23-0089-s02.pdf]

Supplementary Figure S1

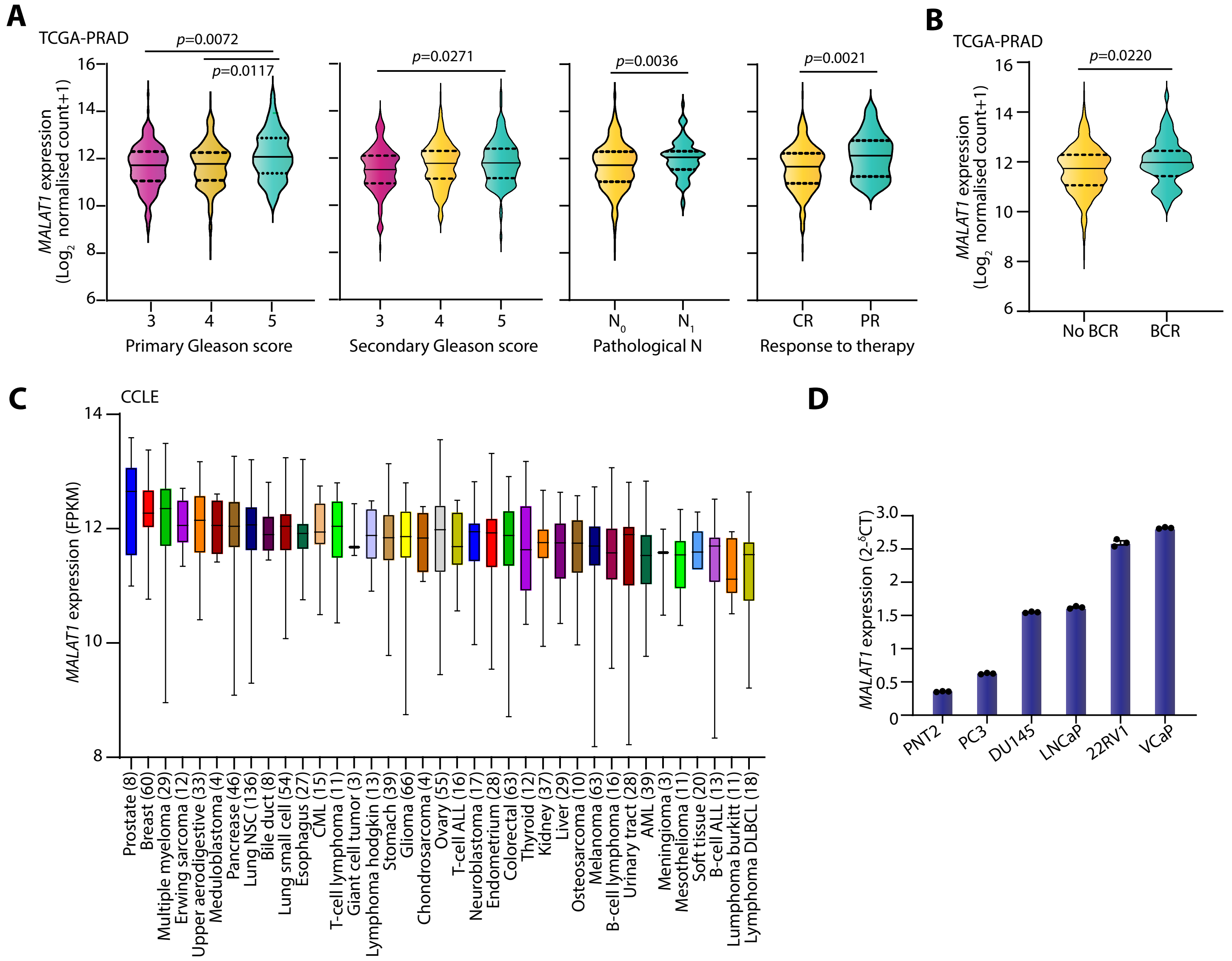

Supplementary Figure S1: ***MALAT1* is upregulated in prostate cancer and positively associates with aggressive clinical phenotype.**

A. Violin plot comparing the *MALAT1* expression in prostate cancer patients (n=499) categorized by varying primary Gleason score, secondary Gleason score, node status, and response to therapy using the RNA-Seq data from the TCGA-PRAD cohort. Expression values are shown as $\log_2$ normalized count+1. Statistical significance was calculated using one-way ANOVA with Dunnett's multiple-comparisons test.

B. Violin plot showing association of *MALAT1* expression with biochemical recurrence in TCGA-PRAD cohort. *MALAT1* transcript values are shown as $\log_2$ normalized count+1.

C. Box plots depicting *MALAT1* expression in multiple human cancer cell lines using RNA-Seq data retrieved from the CCLE database. Expression values are shown as FPKM.

D. Bar plot showing *MALAT1* expression in prostate cancer cell line panel by quantitative PCR. The expression values are represented as $2^{-\delta}$CT. The experiment was performed with n=3 biologically independent samples; data represents mean±SEM.
