# Supplementary material for: Targeting MALAT1 Augments Sensitivity to PARP Inhibition by Impairing Homologous Recombination in Prostate Cancer
Source: Cancer Res Commun. 2023 Oct 9;3(10):2044–61. doi: 10.1158/2767-9764.CRC-23-0089 (PMC10561629; doi:10.1158/2767-9764.CRC-23-0089)
Supplement: Supplementary Figure S2 — MALAT1 positively associates with mesenchymal and stemness markers in prostate cancer patients. [file crc-23-0089-s03.pdf]

Supplementary Figure S2

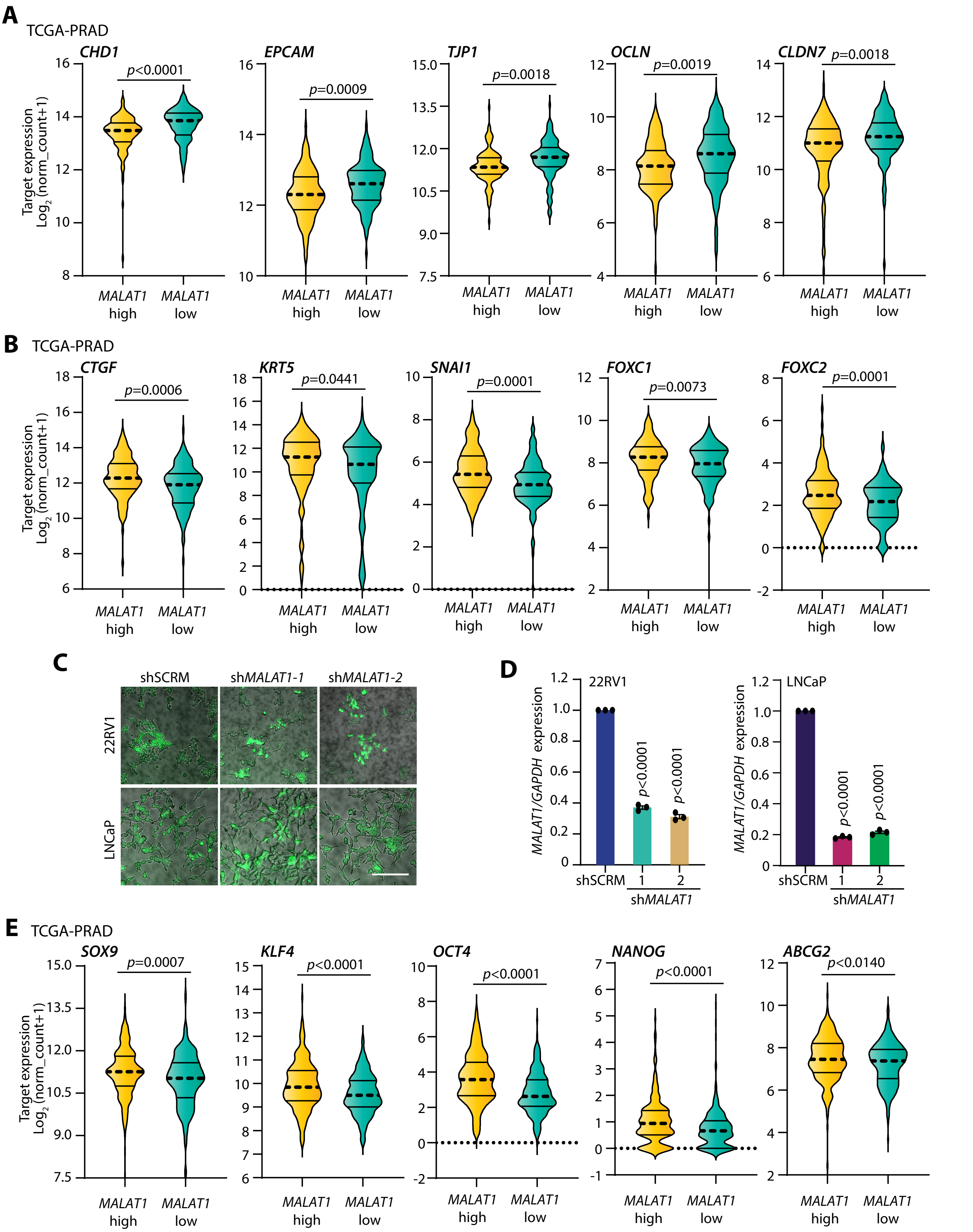

Supplementary Figure S2: *MALAT1* positively associates with mesenchymal and stemness markers in prostate cancer patients.
A. Violin plot depicting expression of epithelial markers in TCGA-PRAD cohort, stratified into "*MALAT1*-high" (n =125) and "*MALAT1*-low" (n = 125) groups based on the quartile-based normalization of *MALAT1* expression.
B. Same as **A**, except for the expression of mesenchymal markers in the TCGA-PRAD cohort.
C. Micrographs showing GFP expression in 22RV1 and LNCaP cells transfected with pLV-eGFP-sh*MALAT1* or -shSCRM.
D. Bar plot depicting expression of *MALAT1* in the same cells as in **c**, measured by quantitative PCR.
E. Same as **A**, except for the expression of stemness markers in the TCGA-PRAD cohort.

The experiments were performed with n=3 biologically independent samples; the data represents mean±SEM. The statistical difference between the samples was calculated using two-tailed unpaired Student's *t*-test for panels **A,B, E** while one-way ANOVA with Dunnett's multiple comparisons was applied for panel **D.**
