# Supplementary material for: Targeting MALAT1 Augments Sensitivity to PARP Inhibition by Impairing Homologous Recombination in Prostate Cancer
Source: Cancer Res Commun. 2023 Oct 9;3(10):2044–61. doi: 10.1158/2767-9764.CRC-23-0089 (PMC10561629; doi:10.1158/2767-9764.CRC-23-0089)
Supplement: Supplementary Figure S3 — MALAT1 regulates the DNA repair pathway in prostate cancer. [file crc-23-0089-s04.pdf]

Supplementary Figure S3

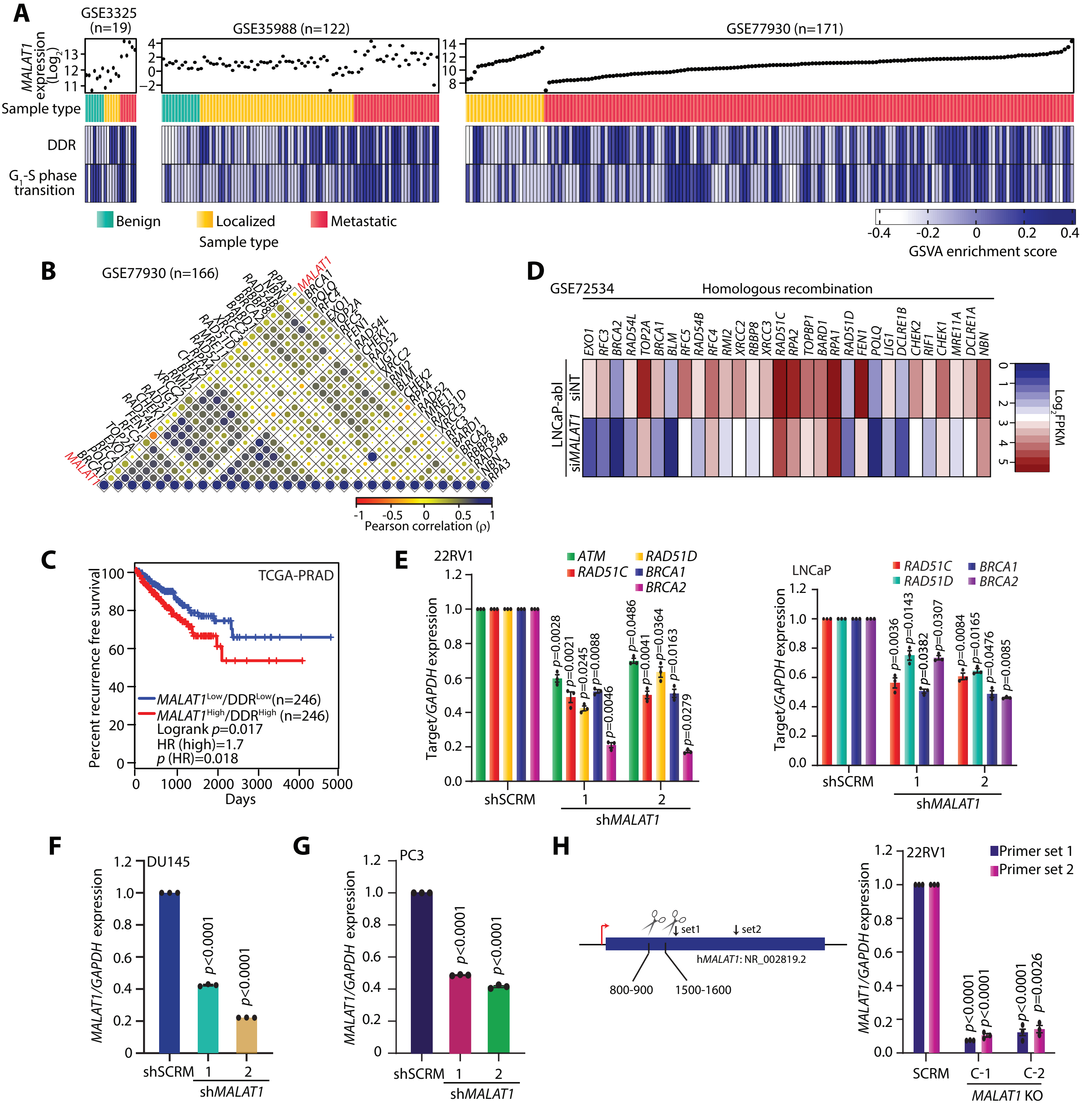

Supplementary Figure S3: ***MALAT1* regulates the DNA repair pathway in prostate cancer.**

A. Heatmap depicting GSVA enrichment scores for DDR and G_1-S transition gene sets significantly enriched (FDR<0.05) in three prostate cancer patient cohorts, namely GSE35988, GSE77930, and GSE3325. The gene set enrichment scores values are expressed by the shades of blue and white. The topmost annotation in the heatmap indicates *MALAT1* expression and the second annotation represents the tumor subtype for the sample.

B. Correlogram representing Pearson correlation coefficient (ρ) between genes associated with DNA repair and *MALAT1* in prostate cancer patient samples from GSE77930 dataset (FDR adjusted, $p<0.05$). Correlation coefficients are expressed by shades of red and blue, and the size of dots is proportional to the strength of the correlation. Representative genes are marked on the sides of the correlogram.

C. Kaplan-Meier curve for relapse-free survival in TCGA-PRAD cohort categorized into two groups according to the median expression of *MALAT1* and DDR genes signature. The red line represents high gene expression, whereas the blue line represents low gene expression. The *p*-value was computed by log-rank test.

D. Heatmap depicting the expression of downregulated DNA repair genes in the LNCaP-abl cells on silencing *MALAT1* as compared to LNCaP-abl siNT. Shades of blue and red represent $\log_2$ fold-change in gene expression.

E. QPCR depicting expression of DDR genes in sh*MALAT1* and shCRM PCa cells.

F. Bar plot depicting expression of *MALAT1* in DU145-sh*MALAT1* and -shSCRM cells by quantitative PCR .

G. Same as **F**, except PC3-sh*MALAT1* and -shSCRM cells.

H. Q-PCR showing *MALAT1* expression in 22RV1-SCRM and 22RV1-*MALAT1*-KO cells.

The experiment was performed with n=3 biologically independent samples; data represents mean±SEM and significance was calculated using one–way ANOVA with Dunnett's multiple comparison test for panel **E**, **F** and **G**.
