# Supplementary material for: Targeting MALAT1 Augments Sensitivity to PARP Inhibition by Impairing Homologous Recombination in Prostate Cancer
Source: Cancer Res Commun. 2023 Oct 9;3(10):2044–61. doi: 10.1158/2767-9764.CRC-23-0089 (PMC10561629; doi:10.1158/2767-9764.CRC-23-0089)
Supplement: Supplementary Figure S4 — MALAT1 depletion restrains cell cycle progression in prostate cancer. [file crc-23-0089-s05.pdf]

Supplementary Figure S4

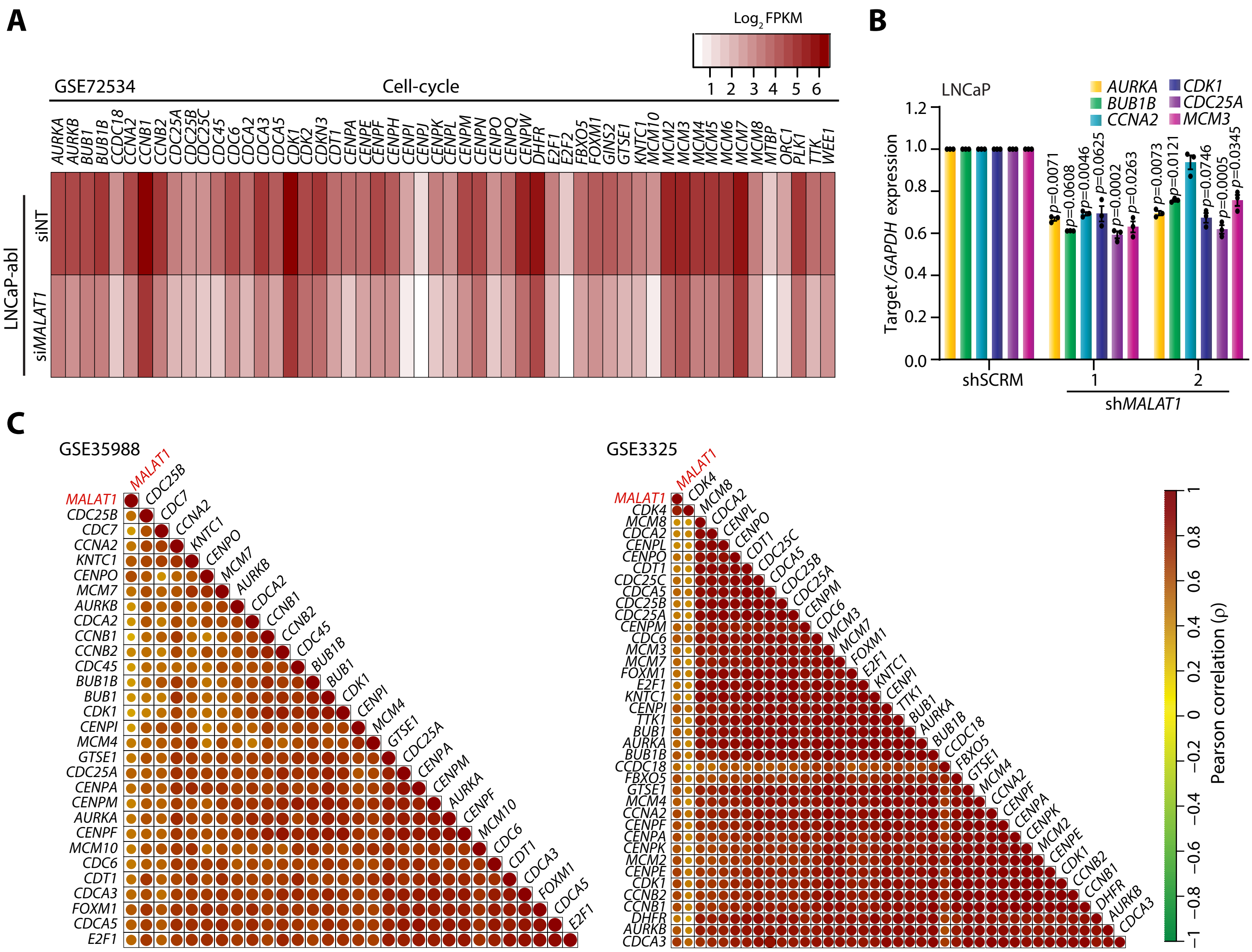

Supplementary Figure S4: *MALAT1* depletion restrains cell cycle progression in prostate cancer.

A. Heatmap depicting differential expression of genes associated with the cell cycle in LNCaP-abl-si*MALAT1* compared to LNCaP-abl-siCTL cells. Shades of red represent $\log_2$ fold-change in gene expression.

B. QPCR depicting expression of cell-cycle markers in LNCaP-sh*MALAT1* and SCRM cells. The experiment was performed with n=3 biologically independent samples; data represents mean±SEM and significance was calculated using one–way ANOVA with Dunnett's multiple comparison test.

C. Correlogram representing Pearson correlation coefficient (ρ) between genes associated with cell cycle and *MALAT1* in prostate cancer patient samples from GSE35988 and GSE3325 dataset (FDR adjusted, $p<0.05$). Correlation coefficients are expressed by shades of green and red, and the size of dots is proportional to the strength of the correlation. Representative genes are marked on the sides of the correlogram.
