# Supplementary material for: Targeting MALAT1 Augments Sensitivity to PARP Inhibition by Impairing Homologous Recombination in Prostate Cancer
Source: Cancer Res Commun. 2023 Oct 9;3(10):2044–61. doi: 10.1158/2767-9764.CRC-23-0089 (PMC10561629; doi:10.1158/2767-9764.CRC-23-0089)
Supplement: Supplementary Figure S5 — MALAT1 does not interact with HR proteins. [file crc-23-0089-s06.pdf]

Supplementary Figure S5

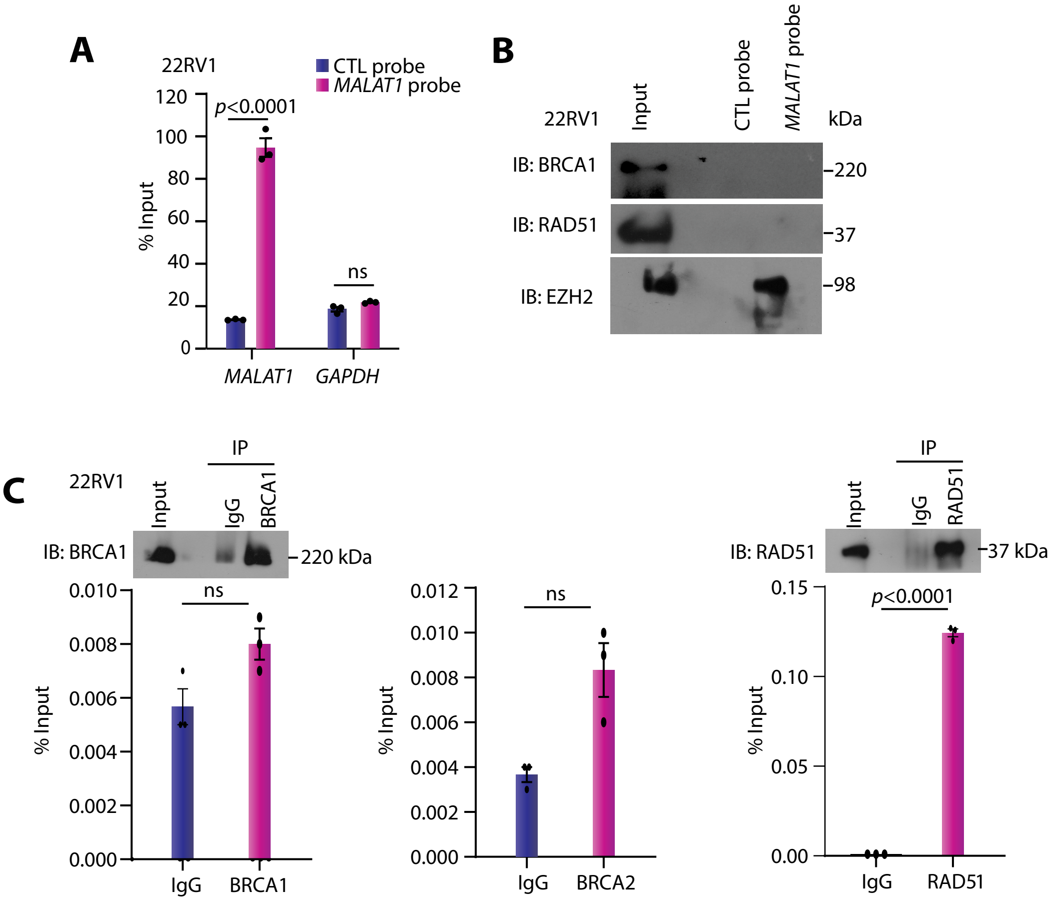

Supplementary Figure S5. *MALAT1* does not interact with HR proteins.

A. Quantitative PCR analysis of ChIRP RNA reveals retrieval of *MALAT1* with its antisense probe but not with the control probes.

B. Immunoblot depicting expression of BRCA1, RAD51 and EZH2 in the same samples as in **A**.

C. RNA immunoprecipitation followed by real-time qPCR analyses demonstrating enrichment of *MALAT1* with target antibody-bound beads in comparison to IgG (control antibody) in 22RV1 cells.
