# Supplementary material for: Targeting MALAT1 Augments Sensitivity to PARP Inhibition by Impairing Homologous Recombination in Prostate Cancer
Source: Cancer Res Commun. 2023 Oct 9;3(10):2044–61. doi: 10.1158/2767-9764.CRC-23-0089 (PMC10561629; doi:10.1158/2767-9764.CRC-23-0089)
Supplement: Supplementary Figure S6 — MALAT1 depletion enhances sensitivity to PARP inhibitors in HR proficient PCa cells. [file crc-23-0089-s07.pdf]

Supplementary Figure S6

A

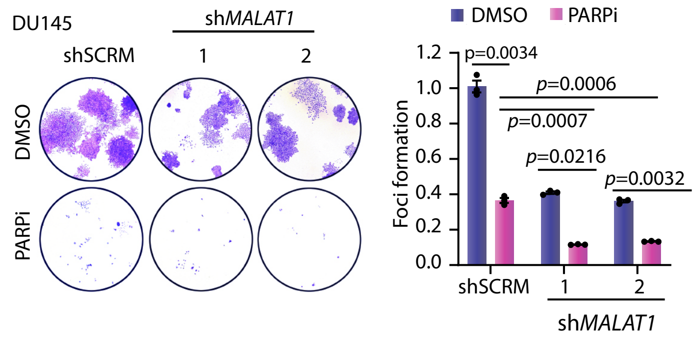

B

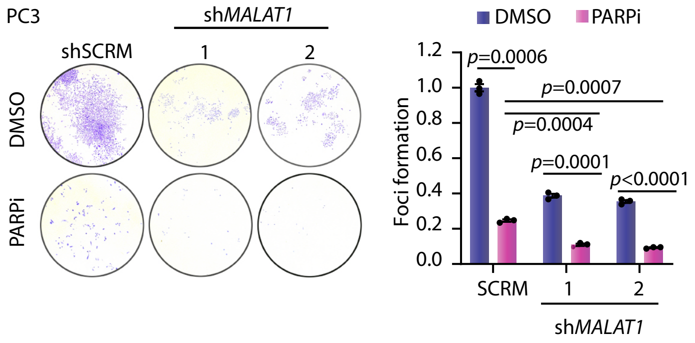

Supplementary Figure S6: *MALAT1* depletion enhances sensitivity to PARP inhibitors in HR proficient PCa cells.
A. Foci formation assay in DU145-SCRM and -sh*MALAT1* cells following treatment with Olaparib (10µM) or vehicle control for 15 days. Inset showing representative images of foci. Bar diagram showing comparative colony number formed by the indicated cells after ten days of culture in serum deprived conditions (right panel).
B. Same as **A**, except PC3-sh*MALAT1* and -shSCRM cells.

The experiments were performed with n=3 biologically independent samples; the data represents mean±SEM and significance was calculated using one–way ANOVA with Dunnett's multiple comparisons test.
