# Supplementary material for: Targeting MALAT1 Augments Sensitivity to PARP Inhibition by Impairing Homologous Recombination in Prostate Cancer
Source: Cancer Res Commun. 2023 Oct 9;3(10):2044–61. doi: 10.1158/2767-9764.CRC-23-0089 (PMC10561629; doi:10.1158/2767-9764.CRC-23-0089)
Supplement: Supplementary Figure S7 — MALAT1 and PARP depletion cooperatively suppress expression of HR genes in HR-deficient as well as HR-proficient PCa cells. [file crc-23-0089-s08.pdf]

Supplementary Figure S7

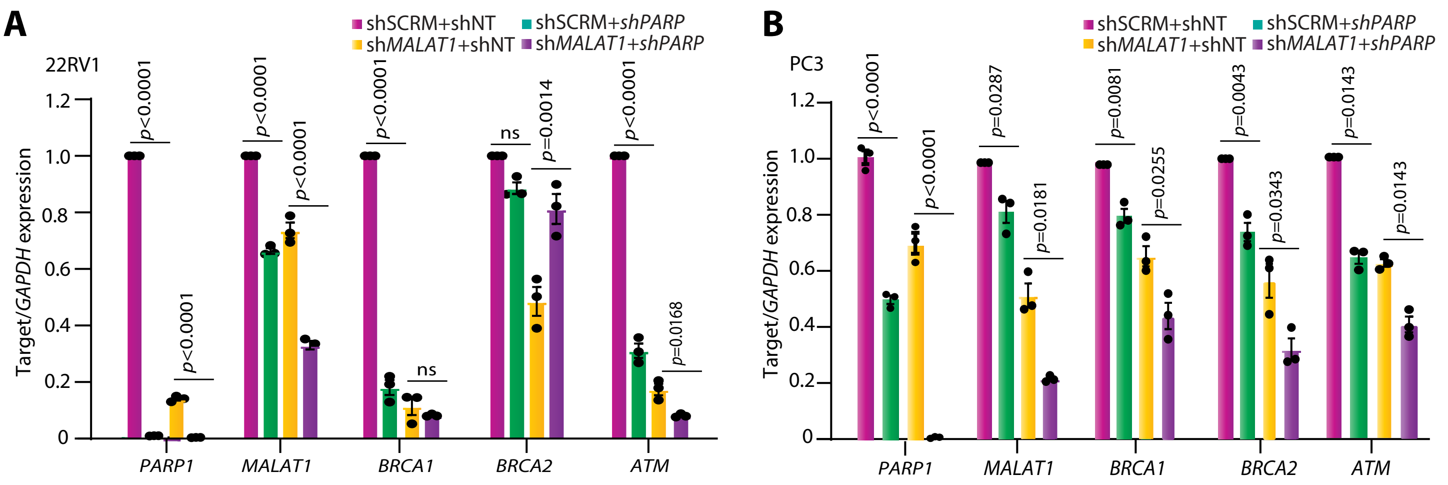

Supplementary Figure S7: *MALAT1* and *PARP* depletion cooperatively suppress expression of HR genes in HR-deficient as well as HR proficient PCa cells.
A. Quantitative PCR depicting relative expression of HR genes in 22RV1-shSCRM and -sh*MALAT1* cells transfected with shRNA against PARP or scrambled control.
B. Same as **A**, except expression of HR genes in PC3-shSCRM and -sh*MALAT1* cells.

The experiments were performed with n=3 biologically independent samples; the data represents mean±SEM and significance was calculated using one–way ANOVA with Dunnett's multiple comparisons test.
